# Targeted UDP-glucose ceramide glucosyltransferase stable overexpression induces a metabolic switch improving cell performance at high cell density

**DOI:** 10.3389/fbioe.2025.1690203

**Published:** 2025-10-13

**Authors:** Marzia Rahimi, Lars K. Nielsen, Jesús Lavado-García

**Affiliations:** ^1^ The Novo Nordisk Foundation Center for Biosustainability, Technical University of Denmark, Lyngby, Denmark; ^2^ Autralian Institute for Bioengineering and Nanotechnology, The University of Queensland, Brisbane, QLD, Australia

**Keywords:** targeted integration, UGCG, cell density effect, HEK293, cell line engineering, virus-like particle (VLP)

## Abstract

**Background:**

Viral vectors such as adeno-associated viruses (AAVs) and virus-like particles (VLPs) are critical tools in gene therapy, typically produced using transient gene expression (TGE). Intensification of TGE processes to high cell densities is hampered by the cell density effect (CDE), characterized by decreased cell-specific productivity as cell density increases. Physiological changes following transfection, particularly reduced glycosphingolipid biosynthesis, have been identified as factors affecting productivity and viability.

**Methods:**

We used a targeted integration approach to generate HEK293SF-3F6 cell lines constitutively or inducibly overexpressing UDP-glucose ceramide glucosyltransferase (UGCG), the precursor enzyme responsible for glycosphingolipid biosynthesis. We evaluated how varying UGCG expression levels influenced cellular metabolism, transfection efficiency, and HIV-1 Gag VLP production.

**Results:**

Constitutive UGCG overexpression triggered a metabolic shift from glycolysis toward mitochondrial fatty acid oxidation. Moderate UGCG expression improved transfection efficiency and enhanced VLP production at high cell densities, while high UGCG expression negatively impacted cellular performance. Inducible UGCG expression further enhanced productivity under high-density conditions, highlighting the advantages of tightly regulated transgene expression.

**Conclusion:**

These findings highlight crucial metabolic adaptations linked to UGCG expression in production cell lines and underscore the value of carefully controlled UGCG expression levels for optimizing viral vector and VLP manufacturing.

## Introduction

Gene therapy has emerged as one of the most promising fields in modern medicine, with growing efforts focused on developing treatments for a wide range of diseases, including blindness, cancer, metabolic disorders, and several other conditions (Bulaklak and Gersbach, 2020; Issa et al., 2023). These therapies function by correcting or replacing defective genes to restore normal cellular processes, offering the potential for long-term therapeutic benefits and, in some cases, curative outcomes (Bulaklak and Gersbach, 2020; Li et al., 2023; Shchaslyvyi et al., 2023). The success of gene therapy depends on the use of efficient and reliable delivery platforms—primarily viral vectors such as adeno-associated viruses (AAVs), adenoviruses (AdVs), and lentiviral vectors (LVs), as well as virus-like particles (VLPs) (Mandalawatta et al., 2024; Ghosh et al., 2020; Ling et al., 2025; Schwartze et al., 2022; Wold and Toth, 2025). These systems facilitate the precise and targeted introduction of therapeutic genetic material into host cells (Li et al., 2023; Ning et al., 2024; Fu et al., 2023). As the number of approved gene therapies continues to rise and clinical pipelines expand, there is a growing need to scale up manufacturing processes to meet global demand.

Currently, the production of viral vectors and VLPs largely relies on transient gene expression (TGE), a method favored for its flexibility and relatively short production timelines. However, large-scale production and achievement of high vector titers remain significant limitations of TGE (Fu et al., 2023). One of the primary challenges associated with TGE is the cell density effect (CDE), a phenomenon observed when cells are transfected at high cell densities, leading to reduced transfection efficiency and cell-specific productivity. Extensive research has been conducted to investigate the underlying mechanisms of CDE, with two major contributing factors proposed. The first is the accumulation of extracellular inhibitory compounds in the culture medium, which interferes with the transfection process and reduces overall efficiency. To overcome this issue, several strategies have been proposed, including exchanging the medium prior to transfection, diluting cultures with fresh medium following transfection, and employing perfusion-based systems that continuously refresh the culture environment to maintain favorable conditions for transfection (Lavado-García et al., 2022; Henry et al., 2004).

The second factor influencing the CDE is the physiological state of the cells during transfection. Using multiplex quantitative proteomic analyses, we have previously demonstrated that several cellular pathways are disrupted at the time of transfection, including those involved in NADP^+^/ADP-ribosyltransferase activity, glycosphingolipid metabolism, and other critical processes. Among these, glycosphingolipid biosynthesis, which is typically upregulated during cell proliferation, has been found to be significantly downregulated following transfection (Lavado-García et al., 2020). Glycosphingolipids are essential for intracellular vesicle trafficking, particularly in facilitating cholesterol transport from the cytosol to the plasma membrane, also functioning as key signaling molecules in various physiological processes (Ishibashi et al., 2013; Wegner et al., 2018a). The enzyme UDP-glucose ceramide glucosyltransferase (UGCG) plays a central role in this biosynthetic pathway by catalyzing the transfer of glucose from UDP-glucose to ceramide, resulting in the formation of glucosylceramide, the precursor to complex glycosphingolipids (Wegner et al., 2018a; Wegner et al., 2018b; D’Angelo et al., 2013; Vukelić and Kalanj-Bognar, 2001; Ryckman et al., 2020). Our previous studies have demonstrated that transient overexpression of UGCG at the time of transfection can enhance transfection efficiency at high cell density (Lavado-García et al., 2021; Pérez-Rubio et al., 2024). However, in these studies, UGCG expression was performed by transient transfection, limiting its broader impact on cellular physiology, as UGCG overexpression itself was affected by the CDE; therefore, its potential for improving transfection efficiency over time could not be fully explored due to the inherent limitations of transient gene expression systems.

To overcome these limitations, stable overexpression of transgenes presents a more robust and reliable alternative. This can be achieved through two main approaches: random integration and targeted integration. In random integration, the transgene is inserted into non-specific genomic locations, which can result in variable expression due to position effects—where the surrounding genomic environment influences gene activity. Additionally, random integration often leads to the insertion of multiple copies of the transgene, contributing to further variability in expression levels among cells (Zeh et al., 2024; Lee et al., 2018). In contrast, targeted integration involves inserting the transgene into a defined genomic locus, allowing for more controlled, predictable and reproducible expression in the generated isogenic clones (Sergeeva et al., 2020; Catalán-Tatjer et al., 2025; Grav et al., 2018). This approach minimizes positional effects, making it particularly suitable for applications that require consistent transgene expression, such as the study of the effect of specific gene overexpression in the developed stable cell lines (Sergeeva et al., 2020; Grav et al., 2018; Yusufi et al., 2017; Pilbrough et al., 2009; Shin et al., 2020).

In this study, we investigated the impact of different stable UGCG overexpression systems on cell physiology, transfection efficiency and VLP production using a targeted integration approach. In the first step, we generated a HEK293SF-3F6 master cell line (MCL) containing a landing pad (LP) at the AAVS1 locus by employing the CRISPR-Cas9 gene editing system. In the second step, we used recombinase-mediated cassette exchange (RMCE) to generate isogenic cell lines that overexpress the UGCG gene either constitutively or upon induction. Lastly, we compared the optimal time and expression levels of the different systems and analyzed cellular changes associated with UGCG overexpression to optimize TGE at high cell densities.

## Results

### Generation of stable UGCG-expressing cell lines

**FIGURE 1:**
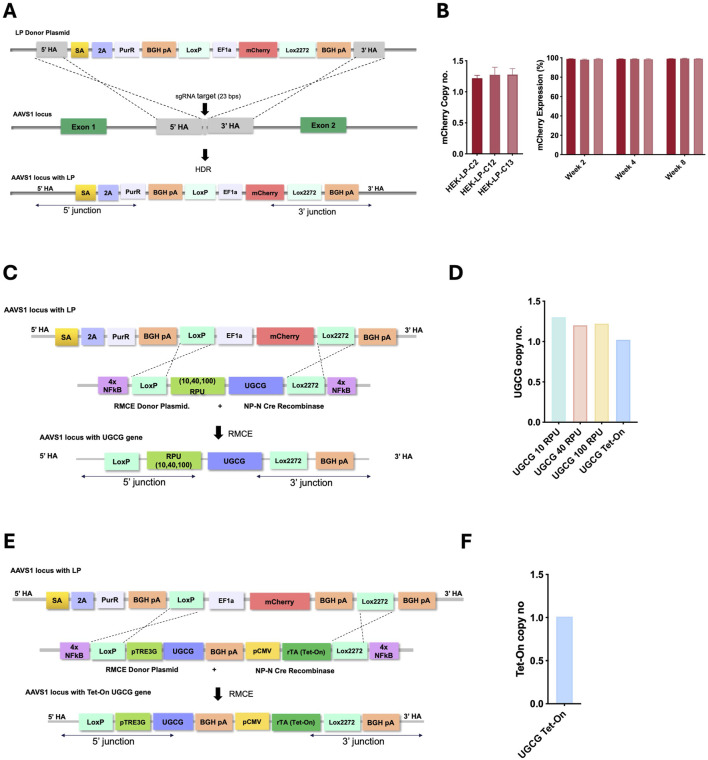
**(A)** Schematic representation of LP integration at the AAVS1 locus via Cas9/sgRNA and homology-directed repair (HDR). **(B)** Copy number analysis of the landing-pad containing clones (left), mCherry expression over 8 weeks of continuous passaging (right). Clone 13 was selected as master cell line for the rest of the work. **(C)** Constitutive system: donor plasmid with synthetic promoters (10, 40, 100 RPU) driving UGCG, flanked by loxP/lox2272. **(E)** Inducible system: donor plasmid with pTRE3G-driven UGCG, inducible by CMV-rtTA; flanked by loxP/lox2272. Both systems include 4× NF-κB response elements and co-transfection with NP-N-Cre for recombination. **(D,F)**. Copy number analysis of the selected clones.

Stable cell lines expressing UGCG were generated through a two-step targeted integration approach. First, HEK293SF-3F6 cells were co-transfected with three plasmids: (i) a donor plasmid containing the LP cassette, (ii) a Cas9-expressing plasmid, and (iii) a plasmid encoding a single-guide RNA (sgRNA) targeting the AAVS1 locus (Supplementary Figure S1). The LP cassette was integrated in the AAVS1 locus between exon 1 and 2 by homology-directed repair (HDR) incorporating the region flanked by Lox sites for further recombination (Figure 1A).

At 72 h post-transfection (hpt), cells underwent puromycin selection for 3 weeks. By the third week, the cell population recovered, reaching viability above 95% and more than 80% of cells showed detectable mCherry fluorescence (Supplementary Figure S2). Single mCherry-positive cells were isolated by fluorescence-activated cell sorting (FACS), and approximately 70% of these isolated clones exhibited correct genomic integration confirmed by junction PCR at both the 5′ and 3′ integration sites. Of the junction-positive clones, 13 were chosen for genomic copy number analysis, with eight confirmed to contain a single integrated LP copy. Three single-copy clones were further characterized for stability and consistently demonstrated high viability, stable growth, and stable mCherry expression throughout 8 weeks of cell passaging (Figure 1B; Supplementary Figure S3). One final clone was selected as the MCL for the second step. To create constitutive UGCG-expressing cell lines, the selected MCL was transfected with a Cre recombinase-expressing plasmid along with independent RMCE donor plasmids encoding UGCG under promoters of varying strengths—10, 40, and 100 RPU (Supplementary Figure S4). This resulted in different cell lines with graded, constitutive UGCG expression (Figure 1C). For inducible UGCG expression, the MCL was similarly transfected with a Cre recombinase plasmid and an RMCE donor plasmid, placing UGCG expression under control of the inducible pTRE3G promoter (Supplementary Figure S4D). The pTRE3G promoter is activated by the reverse tetracycline transactivator (rtTA) in the presence of doxycycline, enabling precise, regulated expression (Figure 1E). To enhance the efficiency of RMCE, nuclear-targeting sequences were incorporated into both the donor and recombinase expression plasmids. Specifically, 4x nuclear factor κB (NF-κB) response elements were introduced at both ends of the transgene cassette to enhance nuclear localization of the donor plasmid. Additionally, an N-terminal nucleoplasmin (NP-N) nuclear localization signal was fused to the Cre recombinase protein to facilitate its entry into the nucleus, as previously described (Shin et al., 2021). These modifications significantly improved RMCE efficiency, resulting in a 40-fold increase in the proportion of mCherry-negative cells, indicating successful cassette replacement (Supplementary Figure S5). Correct cassette replacement was further confirmed by junction PCR at both the 5′ and 3′ integration sites (Supplementary Figure S6). From these validated clones, genomic copy-number analysis identified several clones containing a single integrated copy of UGCG or both UGCG and Tet-On cassettes, for the contitutive and inducible cell lines respectively (Supplementary Figure S7). One of these single-copy clones was then selected for subsequent experiments (Figures 1D,F).

### Growth and metabolic profiles of UGCG-overexpressing cell lines

**FIGURE 2:**
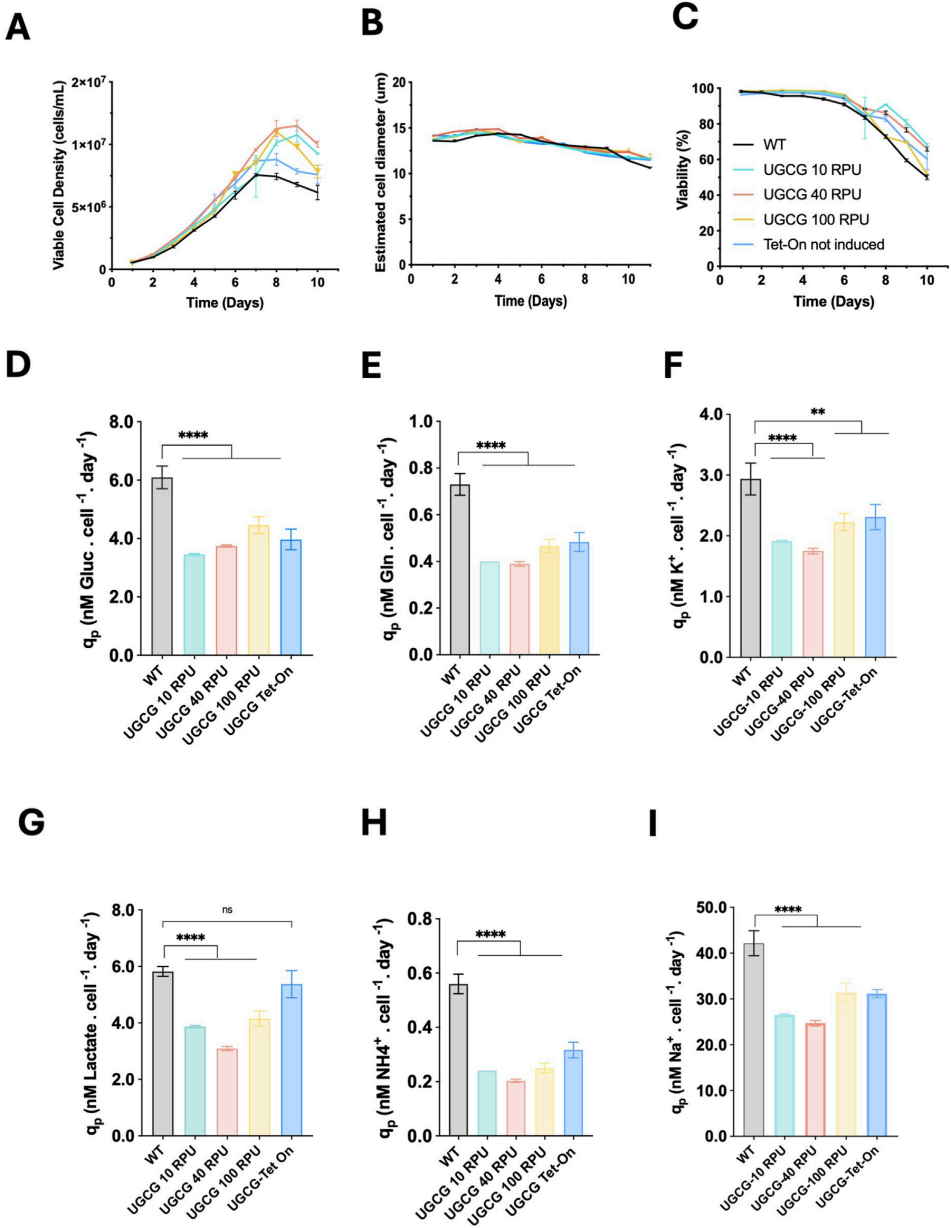
Batch culture performance of generated UGCG stable cell lines. **(A)** Viable cell density (cells/mL). **(B)** Estimated cell diameter (µm). **(C)** Cell viability (%) **(D)** Specific glucose consumption rate. **(E)** Specific glutamine consumption rate. **(F)** Specific potassium uptake rate. **(G)** Specific lactate production rate. **(H)** Specific ammonia production rate. **(I)** Specific sodium accumulation rate. All rates are expressed as nanomoles per cell per day (nM/cell/day). UGCG-Tet-on cell lines were not induced. Data represent the mean ± SD of three biological replicates. Statistical significance was determined using one-way ANOVA and Dunnett’s test; ns: not significant, **p < 0.01, ****p < 0.0001.

To investigate how stable UGCG overexpression affects cell growth and viability, the generated cell lines were cultured under batch conditions for 10 days. All cell lines exhibited exponential growth for the first 3–8 days, followed by a stationary phase, staying in exponential phase 2 days longer than wild-type (WT) which entered stationary phase at day 6 (Figure 2A). Constitutive UGCG-overexpressing cells displayed enhanced growth, achieving a maximum viable cell density (VCD) of approximately 12 × 10^6^ cells/mL. In contrast, WT and non-induced UGCG-Tet-On clones reached lower maximum VCDs, around 7 × 10^6^ cells/mL, under identical culture conditions. Additionally, WT cells experienced a more rapid decline in viability compared to both constitutive and non-induced UGCG-overexpressing cells (Figures 2A–C).

To better understand the metabolic basis of the observed growth differences, metabolites in culture media samples were analyzed daily. Constitutive and non-induced UGCG-overexpressing cell lines showed significantly reduced specific uptake rates of glucose, glutamine, and potassium compared to WT cells. These cell lines also exhibited significantly lower production rates of ammonia and reduced sodium accumulation. Interestingly, lactate production rates were significantly decreased only in constitutive UGCG-overexpressing cells, while non-induced Tet-On cells exhibited lactate production rates similar to WT cells (Figures 2D–I). Collectively, these data indicate a pronounced metabolic shift associated with stable UGCG overexpression under batch culture conditions.

### Extracellular vesicle production in constitutive and inducible UGCG-overexpressing cell lines

To determine whether UGCG overexpression affects the production of extracellular vesicles (EVs), we analyzed EV and VLP concentration and size distribution using nanoparticle tracking analysis (NTA) in cell lines non-transfected and transfected with pGag::eGFP plasmid. Cells were seeded at 0.5 × 10^6^ cells/mL, and EV-containing supernatants were collected when cell densities reached approximately 2 × 10^6^ cells/mL. Analysis of all diffracting particles in non-transfected supernatants revealed EV size distribution and concentration. For experiments involving transfection, cells were transfected at a density of 2 × 10^6^ cells/mL, and samples were harvested 72 h post transfection.

**FIGURE 3:**
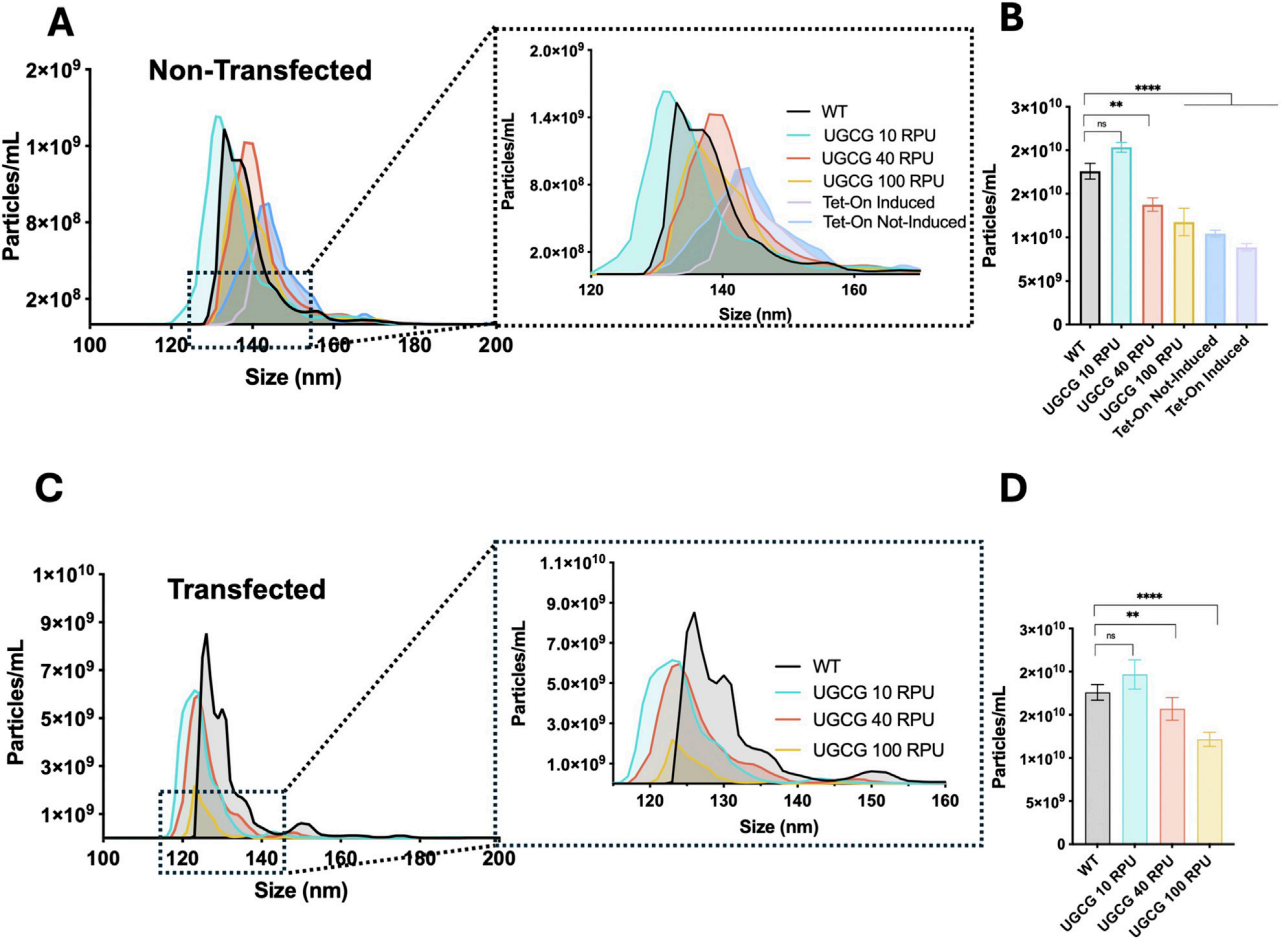
Characterization of extracellular vehicles (EVs) by Nanoparticle Tracking Analysis (NTA). **(A)** Size distribution profile of all diffracting particles (EVs) from the supernatant of non-transfected cell lines cultured at 2 million cells/mL. **(B)** Total particle concentration measured in non-transfected samples. **(C)** Size distribution profile of all diffracting particles (EVs and virus-like particles) from the supernatant of transfected cell lines. **(D)** Total particle concentration 72 hpt transfection. (I) Summary of trends of each studied parameter at 72 hpt compared to wild type behaviour across the different promoter strength. Data represent the mean ±SD of three biological replicates. Statistical significance was determined using one-way ANOVA and Dunnett’s test, ns: not significant, ****p < 0.0001, ***p < 0.001, **p < 0.01 and *p < 0.05.

Under non-transfected conditions, constitutive cell lines expressing UGCG from the weaker promoters (10 and 40 RPU) produced EV concentrations comparable to WT cells. However, cells expressing UGCG from the stronger 100 RPU promoter showed significantly reduced EV concentrations. Similarly, the inducible UGCG Tet-On cell line exhibited decreased EV production upon induction compared to WT (Figure 3B). EV size analysis revealed that constitutive cells driven by the 10 RPU promoter released smaller vesicles on average compared to WT and cells with stronger promoters. In contrast, EVs from Tet-On cells displayed a size distribution comparable to or slightly larger than WT (Figure 3A).

After transfection, all constitutive UGCG-overexpressing cell lines showed reduced total particle concentrations compared to WT cells. The strongest reduction was observed in the UGCG 100 RPU cell line, while moderate reductions occurred in the 10 and 40 RPU cell lines (Figure 3D). Additionally, particle size distributions for all transfected UGCG-expressing cells shifted toward smaller particle sizes relative to WT (Figure 3C). Together, these results demonstrate that stable UGCG overexpression significantly influences both the quantity and size of the particles produced by these cell lines.

### VLP production in constitutive UGCG-overexpressing cell lines

**FIGURE 4:**
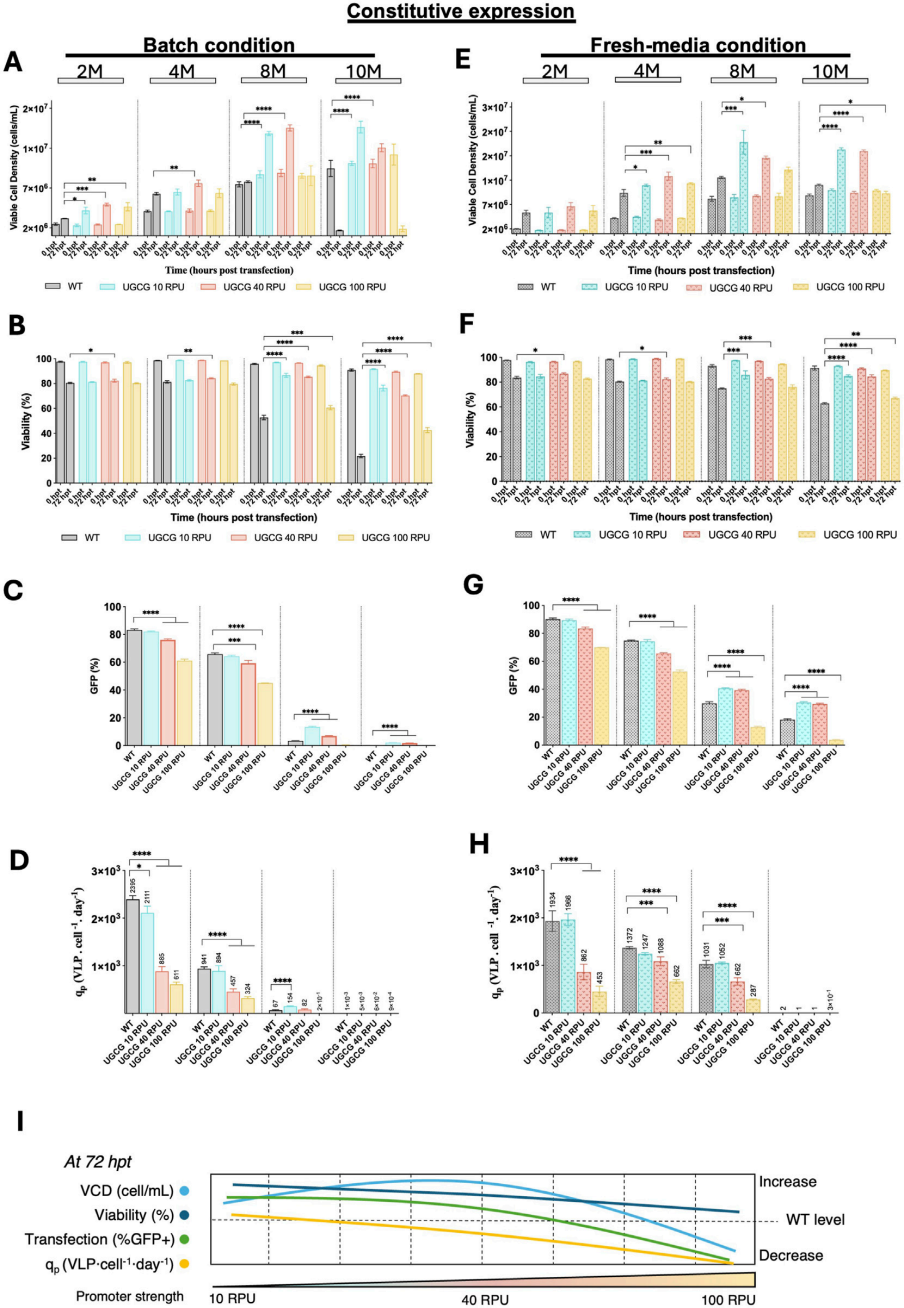
Comparison of cell growth, transfection efficiency and virus-like particle (VLP) specific productivity for the constitutive cell lines in batch and upon fresh media replacement. **(A–D)** Cells were grown in batch. **(E–H)** Cells were transfected after a fresh media replacement. **(A,E)** Viable cell density (cells/mL) measured at different densities (2, 4, 8, and 10 × 10^6^ cells/mL) at the time of transfection and 72 h post-transfection. **(B,F)** Cell viability (%) assessed at the time of transfection and 72 h post-transfection. **(C,G)** Transfection efficiency represented by the percentage of GFP-positive cells. **(D,H)** Specific productivity. **(I)** Summary of the tendencies for the studied parameters in the contitutive UGCG-overexpressing cell lines across different promoter strengths. All experiments were performed in three biological replicates. Statistical significance was determined using one-way ANOVA and Dunnett’s test: ****p < 0.0001, ***p < 0.001, **p < 0.01, *p < 0.05. M: million cells/mL. Bars with pattern represent the conditions transfected after fresh media replacement.

To investigate how UGCG expression levels impact VLP production across different cell densities, WT and constitutive UGCG-expressing cell lines were compared under batch and fresh media replacement conditions. VCD and viability were assessed immediately before and 72 h post-transfection at cell densities of 2, 4, 8, and 10 × 10^6^ cells/mL (Figures 4A,B,E,F). Additionally, transfection efficiency and VLP productivity were measured 72 h after transfection.

Under both culture conditions, UGCG-overexpressing cell lines driven by 10 and 40 RPU promoters displayed enhanced cell growth and higher viability after transfection compared to WT cells, particularly at higher cell densities (8 and 10 × 10^6^ cells/mL). Conversely, cells expressing UGCG under the stronger 100 RPU promoter exhibited either similar or reduced growth and viability relative to WT. At lower cell densities (2 and 4 × 10^6^ cells/mL), transfection efficiency, indicated by GFP expression, was comparable between WT and UGCG 10 RPU cells but significantly reduced in UGCG 40 and 100 RPU cell lines. Notably, at 8 × 10^6^ cells/mL, the UGCG 10 and 40 RPU cell lines demonstrated significantly improved transfection efficiency, showing 4-fold and 2-fold increases in GFP-positive cell populations, respectively, compared to WT cells (Figures 4C,G).

VLP cell-specific productivity (q_p_) in batch cultures was highest in WT cells at low densities (2 and 4 × 10^6^ cells/mL), with all UGCG-overexpressing cell lines initially producing fewer particles. However, at 8× 10^6^ cells/mL density, UGCG 10 RPU cells showed a notable 2-fold increase in VLP productivity compared to WT, while 40 RPU cells exhibited a moderate 1.2-fold increase. UGCG 100 RPU cells did not produce detectable VLPs at this density. A similar productivity pattern emerged in fresh media replacement cultures, where WT cells again had the highest productivity at lower densities. At 8× 10^6^ cells/mL density, the productivity of UGCG 10 RPU cells matched that of WT cells, whereas 40 and 100 RPU lines exhibited lower productivity. At 10× 10^6^ cells/mL, none of the tested cell lines produced measurable VLPs under either culture condition (Figures 4D,H).

### Ceramide supplementation partially alleviates metabolic stress induced by high-level UGCG expression

UGCG catalyzes the initial step in glycosphingolipid biosynthesis, transferring glucose from UDP-glucose to ceramide to produce GlcCer (Ishibashi et al., 2013; D’Angelo et al., 2013; Ryckman et al., 2020). Increased UGCG activity might elevate the demand for ceramide, potentially leading to depletion of intracellular ceramide pools and disruption of lipid homeostasis. To evaluate whether the potential ceramide limitation contributes to the observed impaired cellular performance, we supplemented cultures with exogenous ceramide. C2 ceramide was selected as a soluble, cell-permeable short-chain analog that that can easily permeate cells and integrate into endogenous ceramide metabolic pathways, thereby eliciting similar biological responses to long-chain species without requiring liposomal or specialized delivery systems (Guenther et al., 2008; Van Overloop et al., 2007; Pastore et al., 2016; Bandet et al., 2023). Interestingly, only the cell line expressing UGCG driven by the strongest promoter (100 RPU) showed improvements in transfection efficiency and VLP productivity upon ceramide supplementation (Supplementary Figure S8). This finding suggests that ceramide might be depleted in the cell line bearing the strongest UGCG overexpression, and that this could play a critical role in the stress phenotype observed by high level UGCG expression. However, ceramide supplementation alone did not fully restore cellular functionality, suggesting that additional metabolic or cellular stress mechanisms are involved.

### VLP production in inducible UGCG-overexpressing cell lines

To determine the optimal conditions for doxycycline induction of UGCG expression in inducible Tet-On cells, a rotatable central composite design (RCCD) was employed. In this experimental approach, we examined a range of doxycycline concentrations (10–500 ng/mL) and induction timings (3–6 h post-transfection). The model predictions identified optimal conditions as 81.8 ng/mL doxycycline administered 5.5 h after transfection (Supplementary Figure S9).

**FIGURE 5:**
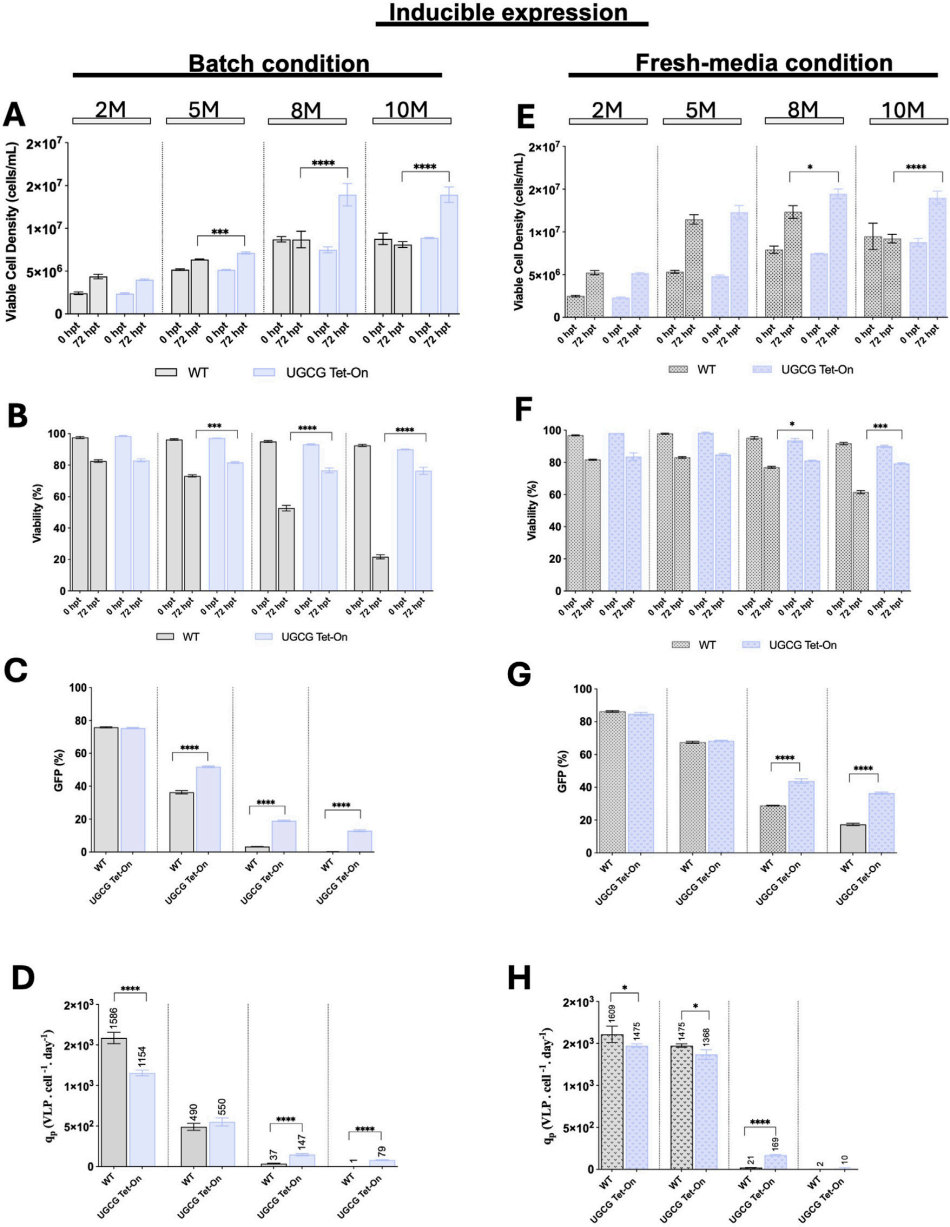
Comparison of cell growth, transfection efficiency and virus-like particle (VLP) specific productivity for the inducible cell line in batch and upon fresh media replacement. **(A–D)** Cells were grown in batch. **(E–H)** Cells were transfected after a fresh media replacement. **(A,E)** Viable cell density (cells/mL) was measured at different densities (2, 5, 8, and 10 × 10^6^ cells/mL) at the time of transfection and 72 h post-transfection. **(B,F)** Cell viability (%) was assessed at the time of transfection and 72 h post-transfection. **(C,G)** Transfection efficiency is represented by the percentage of GFP-positive cells. **(D,H)** Specific productivity. Cell cultures were induced with 81.78 ng/μL doxycycline at 5.5 h post-transfection (hpt). All experiments were performed in three biological replicates. Statistical significance was determined using one-way ANOVA and Dunnett’s test: ****p < 0.0001, ***p < 0.001, **p < 0.01, *p < 0.05. M: million cells/mL. Bars with pattern represent the conditions transfected after fresh media replacement.

Using these optimized induction conditions, WT and inducible UGCG Tet-On cell lines were transfected at various cell densities (2, 5, 8, and 10× 10^6^ cells/mL). Experiments were conducted under both batch and fresh media replacement conditions. Cell growth and viability were monitored immediately before transfection and 72 h post-transfection, while transfection efficiency and VLP productivity were evaluated 72 h after transfection (Figures 5A,B,E,F).

At lower cell densities (2 and 5× 10^6^ cells/mL), WT and inducible UGCG Tet-On cell lines showed comparable growth, viability, transfection efficiency, and VLP productivity under both culture conditions. However, at higher cell densities (8 and 10× 10^6^ cells/mL), inducible UGCG Tet-On cells demonstrated enhanced growth and viability compared to WT. Under batch conditions, inducible cells exhibited significantly increased transfection efficiency—approximately 5.5-fold at 8× 10^6^ cells/mL density and 13-fold at 10× 10^6^ cells/mL density, relative to WT cells (Figure 5C). Correspondingly, VLP cell-specific productivity improved by 4-fold at 8× 10^6^ cells/mL density (Figure 5D). In fresh media replacement cultures, inducible UGCG Tet-On cells also outperformed WT, showing increases in transfection efficiency of 1.5-fold (8× 10^6^ cells/mL) and 2.1-fold (10× 10^6^ cells/mL) (Figure 5G), along with an 8-fold improvement in VLP productivity at 8× 10^6^ cells/mL. Notably, at 10× 10^6^ cells/mL, VLP production was minimal for both WT and inducible cell lines (Figure 5H).

### Proteomic analysis of constitutive UGCG-overexpressing cells (100 RPU) reveals enhanced lipid metabolism and reduced glycolysis

**FIGURE 6:**
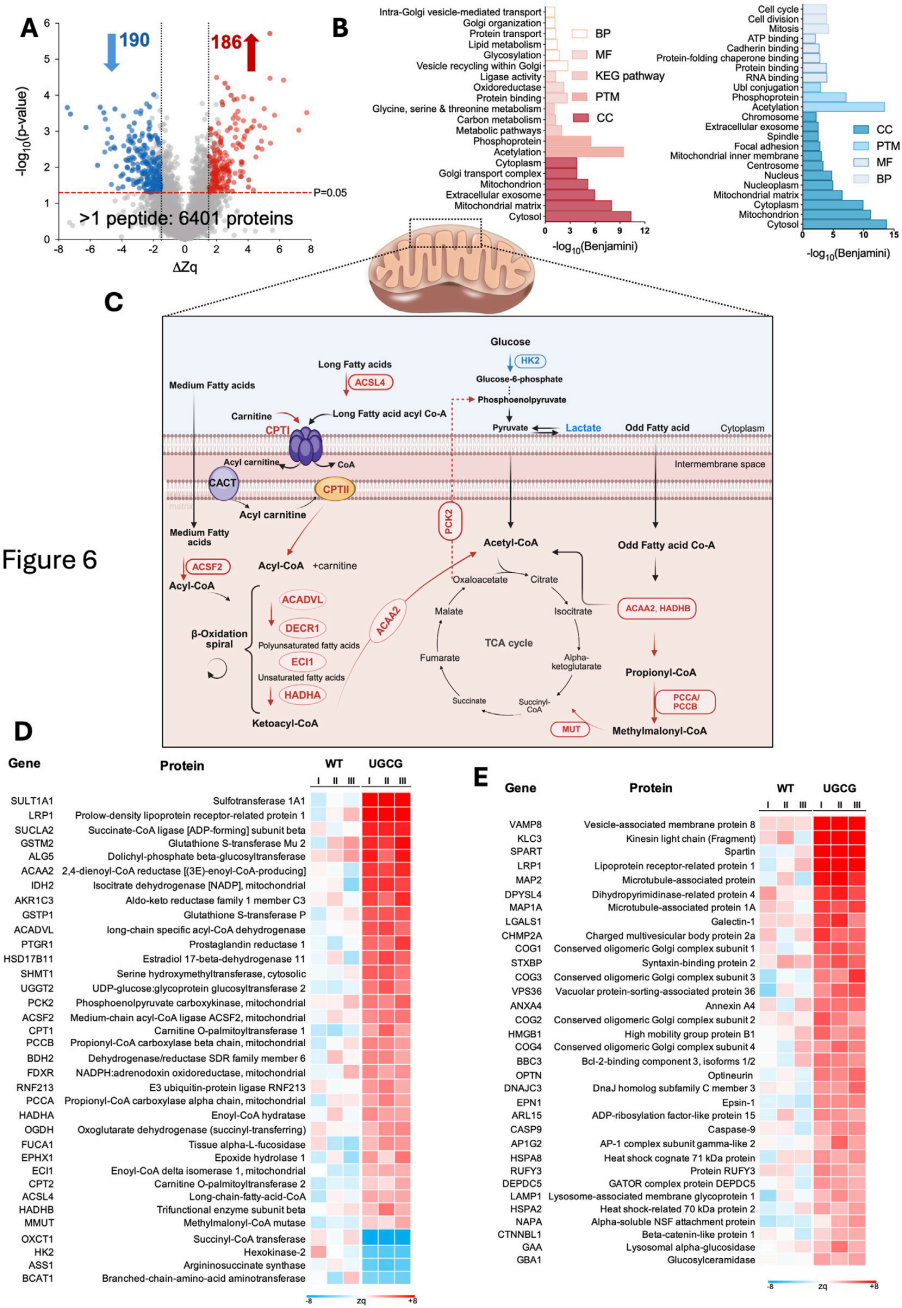
**(A)** Volcano plot showing up- and downregulated proteins in UGCG-overexpressing (100 RPU) vs. wild-type cells. **(B)** Overview of enriched biological processes that are significantly up- and downregulated in UGCG-overexpressing cells. **(C)** Overview of relevant up- and downregulations in mitochondrial fatty acid β-oxidation, glucose metabolism, and their convergence in the TCA cycle. Red and blue labels indicate upregulated and downregulated enzymes respectively. **(D)** Heatmap of differentially expressed proteins involved in metabolic pathways. **(E)** Heatmap of differentially expressed proteins associated with vesicle trafficking, autophagy, lysosomal function, and membrane dynamics. I, II, III represent the three biological replicates.

The UGCG 100 RPU cell line was selected for detailed proteomic analysis due to its pronounced phenotypic differences compared to WT and lower-strength constitutive cell lines. Specifically, the 100 RPU cells exhibited significantly reduced EV and VLP production, alongside markedly decreased transfection efficiency. To investigate the molecular mechanisms underlying these distinct phenotypes, we performed multiplexed quantitative proteomic analyses comparing UGCG 100 RPU cells with WT cells. In total, 8,667 proteins were identified, whereof 6,401 were quantified by more than one peptide (Supplementary Table S1). Significantly altered proteins were defined as those exhibiting ΔZq values ≥1 (upregulated) or ≤ −1 (downregulated), with a statistical threshold of p-value <0.05 (Figure 6A).

Functional enrichment analysis revealed that upregulated proteins were primarily localized to the cytosol, mitochondrial matrix, and extracellular exosomes. These proteins were involved in lipid and amino acid metabolism, and pathways related to Golgi function and vesicle-mediated transport (Figure 6B, left). Downregulated proteins were primarily involved in processes related to cell division, cell cycle progression, mitosis, ATP binding, cadherin binding, and protein folding chaperone activity (Figure 6B, right). Based on observed differences in metabolite profile and vesicle production in UGCG 100 RPU cells, we further analyzed proteins involved specifically in metabolic processes and vesicle trafficking. We identified 35 significantly upregulated metabolic proteins (Figure 6D). Many of these proteins were enzymes involved in the activation and transport of long-chain fatty acids, indicating enhanced mitochondrial fatty acid import. Consistent with this finding, several key β-oxidation enzymes also showed increased expression, supporting an elevated capacity for mitochondrial fatty acid catabolism and energy production. Furthermore, PCK2, a key enzyme involved in gluconeogenesis (Zhang et al., 2024), was significantly upregulated, whereas the glycolytic enzyme HK2 (Roberts and Miyamoto, 2015) was downregulated in UGCG-overexpressing cells (Figure 6C).

In addition to metabolic proteins, several key components involved in vesicle fusion and intracellular trafficking were markedly upregulated (Figure 6E; Supplementary Table S1). Proteins involved in endosomal sorting and multivesicular body formation, including CHMP2A (an ESCRT-III component facilitating membrane scission) (Bertin et al., 2020; Alqabandi et al., 2021) and VPS36 (an ESCRT-II subunit involved in cargo recognition and sorting) (Im and Hurley, 2008), were also significantly upregulated. Lysosomal proteins also displayed increased expression (Supplementary Table S1).

Furthermore, proteins related to autophagy, ATG16L1, like OPTN, a selective autophagy receptor (Xu et al., 2022; Gammoh, 2021), were similarly upregulated.

### Proteomic analysis of transient and induced UGCG overexpression revealed upregulation of key proteins involved in vesicle trafficking and membrane remodelling

**FIGURE 7:**
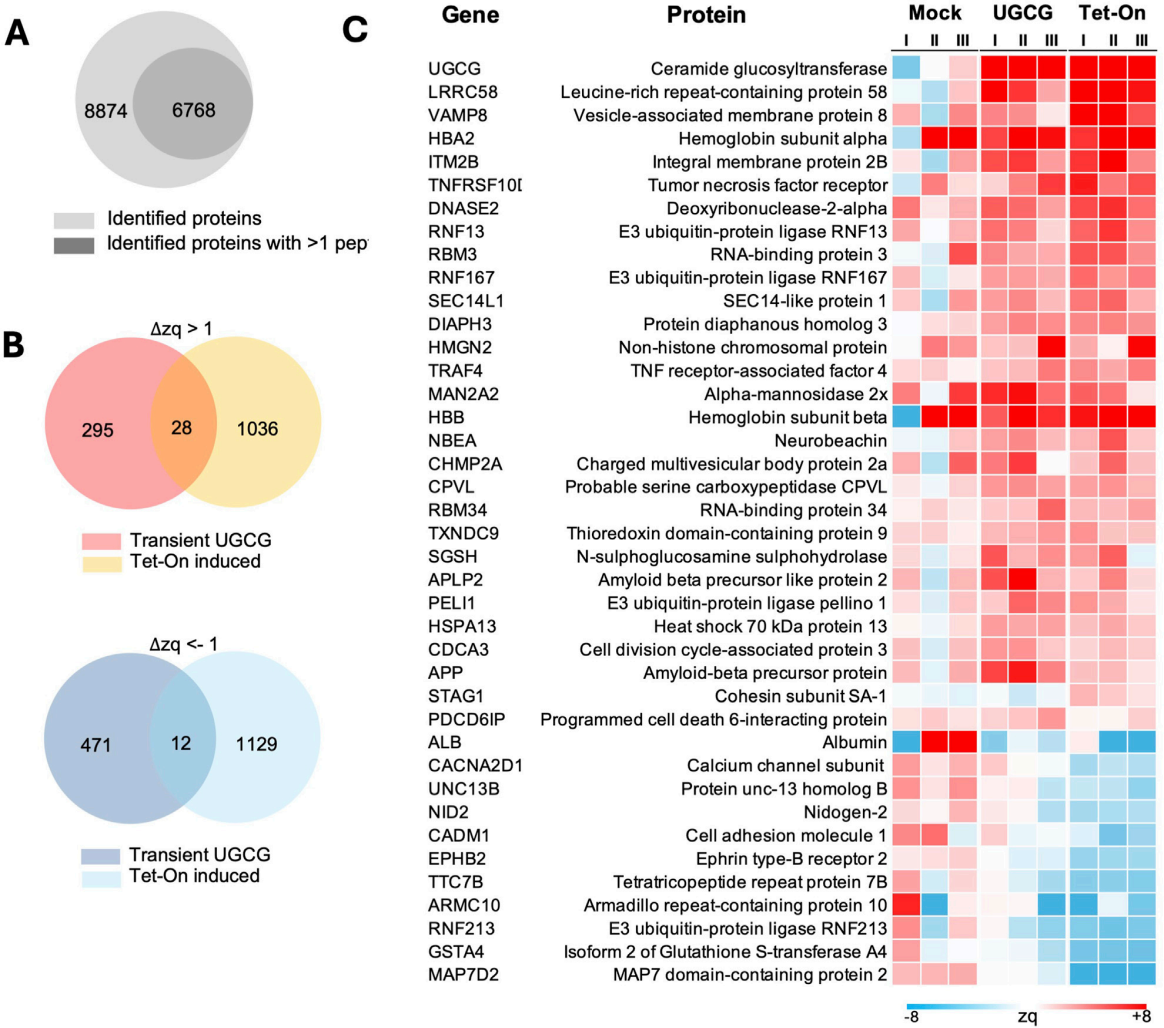
Proteomic analysis of transient and Tet-On-induced UGCG expression at 72 h post-transfection. **(A)** Venn diagram showing the total number of proteins identified (8,874), and the subset identified with more than 1 peptide (6,768). **(B)** Venn diagrams displaying the overlap of upregulated ( ∆ Zq > 1) and downregulated $\left(∆\right.$ Zq < −1) proteins in transiently transfected versus inducibly expressed UGCG conditions. **(C)** Heatmap of all shared protein between transient and Tet-On UGCG that are up- and downregulated. I, II, III represent the three biological replicates.

Since transient transfection (Pérez-Rubio et al., 2024) and Tet-On-induced UGCG overexpression showed the best results in terms of transfection efficiency and cell-specific productivity at high cell densities, we analyzed the proteome alteration in both conditions to identify the potential molecular cause for the improvement. To compare proteomic alterations triggered by transient versus inducible UGCG overexpression, we performed quantitative proteomic analyses at 72 h following either transient transfection or Tet-On–induced expression. Mass spectrometry analysis identified a total of 8,874 proteins, of which 6,768 were quantified with more than one peptide (Figure 7A; Supplementary Table S1). Differential protein expression was analyzed by comparing each UGCG-overexpressing condition (transient and Tet-On) separately against WT cells transfected with a mock plasmid. Significantly altered proteins were defined based on ΔZq values, using thresholds of ΔZq ≥1 for upregulation and ΔZq ≤ −1 for downregulation. Our analysis identified both distinct and overlapping sets of altered proteins between transient and Tet-On–induced UGCG overexpression. Among upregulated proteins, 295 were uniquely elevated following transient expression, 1,036 were specifically increased in Tet-On–induced cells, and only 28 were commonly upregulated under both conditions (Figure 7B, top). Conversely, for downregulated proteins, 471 were uniquely reduced after transient expression, 1,129 were specifically decreased following Tet-On induction, and only 12 proteins were consistently downregulated across both experimental approaches (Figure 7B, bottom). Both expression approaches resulted in increased abundance of key proteins linked to membrane remodelling and vesicle trafficking, notably vesicle fusion regulators, essential ESCRT machinery, and proteins supporting Golgi and post-Golgi membrane transport (Supplementary Table S1). As anticipated, UGCG enzyme itself was elevated, potentially affecting membrane composition and trafficking dynamics (Figure 7C). Conversely, proteins associated primarily with general cellular maintenance, adhesion, synaptic activity, and extracellular matrix integrity exhibited significant downregulation.

### Quantification of UGCG-DYKDDDDK levels across expression systems

To better understand the relationship between UGCG expression levels and the observed effects on metabolism, cellular physiology, transfection efficiency, and VLP productivity, we quantified UGCG expression across transient, constitutive, and inducible systems. Samples from each condition were harvested at equivalent densities (10 × 10^6^ cells/mL per condition), lysed, and UGCG-DYKDDDDK protein levels were determined using a DYKDDDDK-specific ELISA.

**FIGURE 8:**
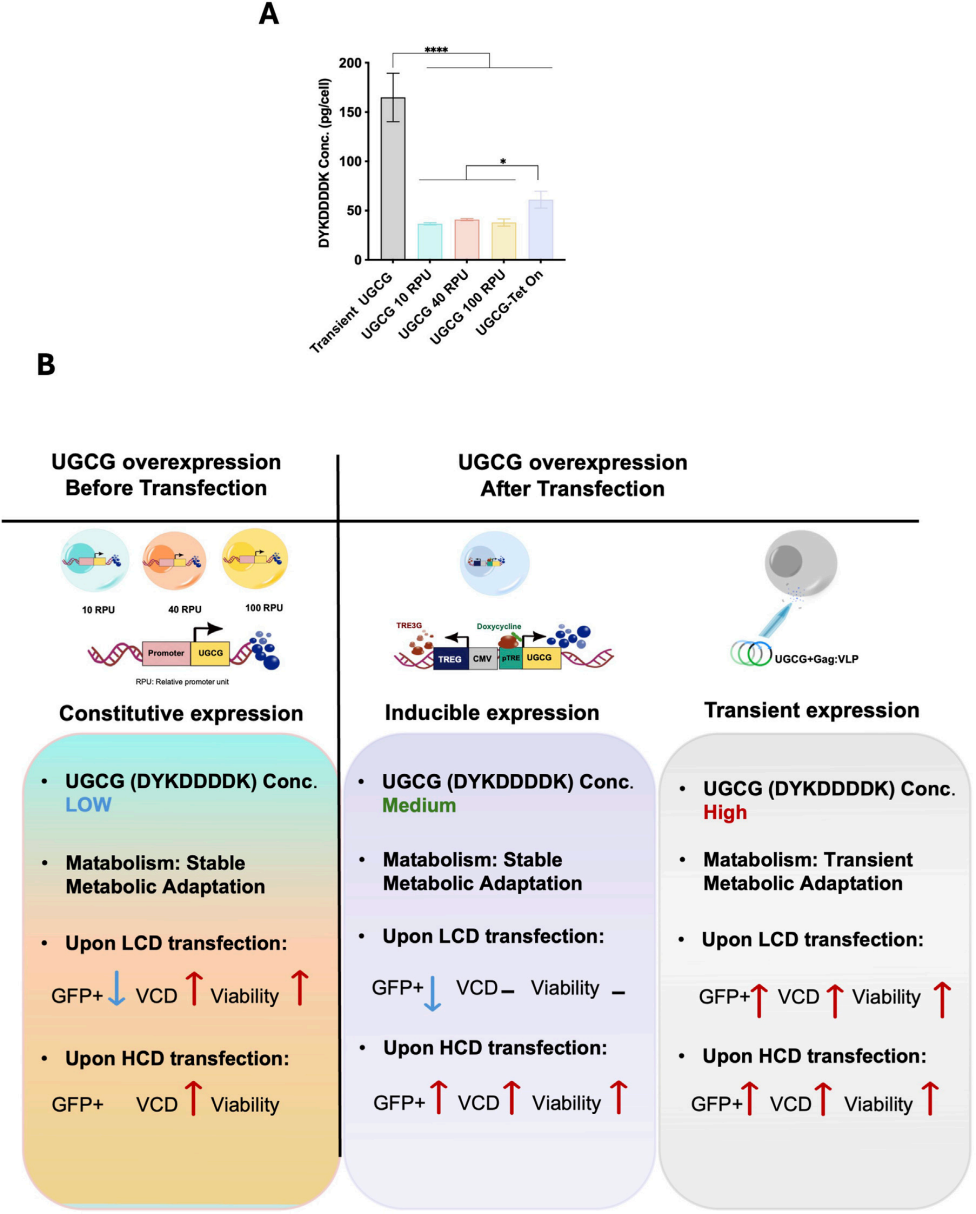
**(A)** Quantification of UGCG expression across constitutive, inducible, and transient overexpression systems. Data represent the mean ± SD from three biological replicates. Statistical significance was determined using one-way ANOVA followed by Dunnett’s test (****p < 0.0001, *p < 0.05). **(B)** Schematic overview of UGCG overexpression strategies and their impact on cell performance. M: million cells, LCD: low cell density, HCD: high cell density, VCD: viable cell density, Pro: promoter. Blue text and downward arrows indicate low expression; green text indicates medium expression; red upward arrows indicate high expression. Transient transfection data are from Pérez-Rubio et al. (2024).

Transiently transfected cells exhibited the highest UGCG protein expression, reaching approximately 165 pg/cell. In comparison, stable constitutive cell lines had substantially lower expression, ranging from 36 to 41 pg/cell, representing approximately a 4-fold reduction compared to transient transfection. Notably, differences in promoter strength (10, 40, and 100 RPU) within stable constitutive cell lines resulted in minimal variation in UGCG protein expression, indicating limited sensitivity of stable UGCG expression to increasing promoter strength under these conditions. Following doxycycline induction (81.8 ng/mL), inducible Tet-On cells expressed intermediate UGCG protein levels (approximately 61.2 pg/cell), higher than constitutive lines but lower than transient expression (Figure 8A).

As indicated by the expression levels, the effects leading to improvement of cell performance at high cell density appear to be more pronounced with higher UGCG expression levels and stable metabolic adaptation appear to lead to a change in metabolism reducing their productivity.

## Disscussion

### Cell line generation

In this study, we employed a targeted integration strategy combining CRISPR-Cas9 with RMCE to generate stable cell lines overexpressing the UGCG enzyme. By precisely inserting the UGCG transgene into the well-characterized genomic safe-harbor locus AAVS1, we aimed to minimize gene expression variability due to position effects, a common challenge associated with random integration approaches (Zeh et al., 2024; Lee et al., 2018; Grav et al., 2018). This targeted approach enabled us to directly attribute the observed phenotypic changes to UGCG overexpression rather than to clonal differences or genomic context. Indeed, we observed a clear correlation between the strength of the promoter driving UGCG expression and cellular phenotypes, specifically reductions in EV release, VLP production, and transfection efficiency at low cell density (Supplementary Figure S9E). This relationship confirms that the observed phenotypes are directly dependent on UGCG expression levels, reinforcing the importance of precise genomic integration.

We initially generated an MCL containing a LP with an mCherry reporter gene integrated into the AAVS1 locus using CRISPR-Cas9. The cells exhibited stable growth and consistent mCherry expression throughout 8 weeks of continuous passaging. Subsequently, we utilized RMCE to replace the LP cassette with the UGCG transgene cassette. Although RMCE is precise and efficient, it typically suffers from low recombination efficiency, especially with larger transgenes or multiple simultaneous insertions (Shin et al., 2021). To overcome this limitation, we employed an optimized nuclear-targeting strategy previously described by Shin et al. (Shin et al., 2021), who demonstrated that including a nucleoplasmin (NP) nuclear localization signal (NLS) in the Cre recombinase plasmid and a nuclear factor-κB (NF-κB) DNA nuclear-targeting sequence (DTS) in the donor plasmid significantly increased recombination efficiency (up to 8.1-fold). Adopting this approach, we fused the NP-NLS to Cre recombinase and incorporated 4x NF-κB DTS into our UGCG expression cassette, achieving an approximately 40-fold increase in RMCE efficiency for single-cassette replacement.

Altogether, our targeted integration and optimized RMCE strategy allowed efficient and rapid generation of stable UGCG-overexpressing cell lines. This approach provided a robust experimental system to thoroughly investigate the metabolic and phenotypic impacts of UGCG expression described in subsequent sections.

### Stable UGCG expression causes permanent metabolic changes that impair TGE at low cell density but enhance performance at high cell density, mitigating the cell density effect

In this study, we explored how stable UGCG overexpression influences cellular physiology, EV production, transfection efficiency, and VLP productivity. Our results demonstrate a clear negative correlation between the level of constitutive UGCG overexpression and transient transfection performance at low cell density. Specifically, stronger promoter-driven UGCG expression (100 RPU) was consistently associated with significant impairments in transfection efficiency, EV secretion, and VLP productivity (Supplementary Figure S9E). These impairments might be partially attributed to potential ceramide depletion resulting from elevated UGCG enzymatic activity. While supplementation with exogenous ceramide appeared to partially restore transfection efficiency and VLP productivity in these cells, it was insufficient to fully recover overall cellular functionality, suggesting that additional metabolic stress mechanisms may also contribute to the observed phenotype. Further analyses of metabolic and proteomic profiles revealed adaptive responses associated with, or triggered by, stable UGCG overexpression.

Notably, we observed a distinct metabolic shift characterized by decreased glycolytic activity and increased indicators of mitochondrial fatty acid oxidation. This reprogramming was reflected in the upregulation of fatty acid transporters and β-oxidation enzymes (e.g., ACSL4, CPT1, ACADVL, and HADHA) (Houten and Wanders, 2010; Houten et al., 2016; Sklirou et al., 2021; Wanders et al., 2010), together with downregulation of glycolytic processes, including reduced glucose uptake, lower lactate production, and decreased HK2 expression. These findings indicate that cells might be responding to a likely altered lipid homeostasis by enhancing fatty acid catabolism and reducing glycolytic metabolism.

The metabolic shifts observed here align with, yet differ from, previous studies, emphasizing context-dependent effects. For example, Schömel et al. reported enhanced energetic metabolism in UGCG-overexpressing breast cancer cells, characterized by increased glycolysis and mitochondrial oxidative activity (Schömel et al., 2020). In contrast, Wegner et al. found suppressed energy metabolism, indicated by reduced mitochondrial respiration and glycolysis, in non-cancerous liver cells overexpressing UGCG (Wegner et al., 2021). Furthermore, Schömel et al. demonstrated enhanced glutamine metabolism and oxidative stress responses following UGCG upregulation, underscoring the complexity and cell-type specificity of UGCG-induced metabolic adaptation (Schömel et al., 2019). This underlines the importance of characterizing the effects of UGCG-overexpression in cell lines cultured in controlled environments and used for the biomanufacturing of complex biologics, since the observed effects cannot be inferred from cell types growing in tissue-contexts.

Our results also highlight significant alterations in vesicle trafficking and autophagy pathways induced by UGCG overexpression. The upregulation of key proteins involved in vesicle fusion and trafficking, such as VAMP8, STXBP, and subunits of the conserved oligomeric Golgi (COG) complex (Huang et al., 2021; Wang and Diao, 2022; Blackburn et al., 2019), indicates enhanced vesicle formation and trafficking, likely as a cellular strategy to manage excess lipid accumulation. Additionally, elevated expression of lysosomal markers (LAMP1, GAA, GBA1) (Cheng et al., 2018; Eskelinen, 2006; Ward et al., 2016), suggests increased vesicular sorting and lysosomal degradation activity. Concurrent upregulation of autophagy-related proteins (ATG16L1, OPTN) (Xu et al., 2022; Gammoh, 2021) further supports the activation of selective autophagic mechanisms aimed at degrading excess lipid cargo or damaged organelles.

This association between altered UGCG expression, sphingolipid metabolism, and autophagy activation is consistent with findings in other biological contexts. For instance, Turathum et al. observed increased autophagy linked to abnormal sphingolipid metabolism involving elevated UGCG activity in granulosa cells of women with endometriosis (Tu et al., 2022). Similarly, Liu et al. identified UGCG among sphingolipid metabolism-related genes implicated in autophagy during sepsis (Wu et al., 2025). Although these studies were conducted in significantly different cellular contexts, their findings parallel our observations, collectively suggesting that UGCG might be driving disruptions in lipid metabolism that can broadly trigger autophagy as a protective cellular response.

Interestingly, while cells expressing UGCG driven by the strongest promoter (100 RPU) exhibited the poorest overall performance, cell lines with moderate and low UGCG expression demonstrated significantly improved performance. Specifically, at higher cell densities, these cells showed up to a 4-fold increase in transfection efficiency and up to a 2-fold improvement in VLP productivity compared to WT cells. This enhanced performance might suggest an optimal balance between sufficient glycosphingolipid synthesis to support membrane remodelling and vesicle trafficking required for efficient VLP assembly and release, without placing excessive demands on cellular resources. In contrast, very high UGCG expression could impose a substantial metabolic burden, diverting ATP, lipid precursors, and trafficking machinery toward UGCG synthesis and processing rather than VLP production. Cellular energy may also be redirected to handling and trafficking potential excess glycosphingolipids. However, a detailed lipidomic analysis would be required to draw definitive conclusions regarding ceramide and other glycosphingolipid profiles. The proteomic analyses provide indirect evidence of metabolic and trafficking changes but do not establish causality, and further functional studies would be needed to validate specific pathways. Furtermore, the study was conducted in HEK293-derived cell lines, and results may not directly translate to other production systems or cell types.

Additionally, elevated expression of CHMP2A and VPS36, essential components of the ESCRT-III and ESCRT-II complexes known to be involved in the assembly and budding of HIV VLPs (Effantin et al., 2013; Wang et al., 2023), respectively, might represent compensatory cellular mechanisms to support and enhance VLP production. Under high-density culture conditions, cells typically encounter nutrient limitations, metabolic byproduct accumulation, and increased cellular stress. Moderate and low levels of UGCG overexpression, combined with enhanced CHMP2A and VPS36 expression, could enable cells to effectively cope with these stressors. Such adaptive responses likely help stabilize cellular membranes, facilitate essential signaling processes, and provide structural lipids without exceeding the cells' metabolic capacity.

### Transient, inducible and stable expression dynamics

In this study, we also generated an inducible Tet-On system designed to finely regulate UGCG expression. Our results demonstrated that the optimized induction conditions enhanced cellular performance, particularly at high cell densities (8 × 10^6^ cells/mL) in batch cultures. Specifically, inducible UGCG-overexpressing cells exhibited a 5.5-fold increase in transfection efficiency, a 4-fold improvement in VLP productivity, and improved cell growth post-transfection relative to WT cells. This superior performance likely reflects the benefit of dynamically controlling UGCG expression, balancing glycosphingolipid synthesis with the metabolic constraints and cellular stresses. Surprisingly inducible UGCG cells displayed reduced glucose and glutamine uptake even in the absence of doxycycline induction (Figures 2D,E), a metabolic profile resembling that observed in constitutive UGCG-overexpressing cell lines. This unexpected finding could indicate low-level (leaky) expression from the inducible promoter, a well-documented issue with inducible systems (Ali Hosseini Rad et al., 2020; Roney et al., 2016; Berens and Hillen, 2003; Kallunki et al., 2019).

Comparing transient, inducible, and stable UGCG expression systems, transient expression showed the highest transfection efficiency (Lavado-García et al., 2021; Pérez-Rubio et al., 2024) due to high but short-lived UGCG protein expression (∼165 pg/cell), causing significant yet transient metabolic and physiological adjustments. Inducible cells offered intermediate UGCG levels (∼61 pg/cell), balancing metabolic stress and productivity, while stable constitutive expression at moderate-to-low promoters (36–41 pg/cell) provided improved performance (Figure 8B).

In summary, our findings highlight the critical importance of precisely controlling UGCG expression levels. Moderate UGCG overexpression optimizes cellular performance by potentially balancing glycosphingolipid biosynthesis and metabolic capacity, whereas excessive UGCG expression might disrupt lipid homeostasis, trigger metabolic stress, and impair essential cellular functions. Further studies should explore detailed lipidomic profiles to elucidate specific lipid changes driven by UGCG expression.

### Biomanufacturing potential of UGCG-overexpressing cell lines

HEK293 cells are the industry standard for manufacturing of viral vectors that rely on transient gene expression because they are easy to transfect, highly permissive to viral replication, and support high productivity of viral particles. They are well characterized, widely used under GMP conditions, and have regulatory acceptance for clinical applications. In addition, their genome harbours the adenoviral genes E1a and E1b, essential for AAV production (Tan et al., 2021). Building on the well-established advantages of HEK293 as a viral vector manufacturing host, we sought to further enhance its performance through targeted metabolic engineering to mitigate the CDE and improve cell performance at HCD. UGCG-overexpressing cell lines, particularly those engineered with inducible Tet-On systems or moderate UGCG expression, offer a new platform for TGE-based biologics production, like VLPs at high cell densities. It has shown to alleviate the CDE, shedding some light into a promising metabolic engineering approach for viral vector production.

Although inducible UGCG-expressing cells offer improved transfection efficiency and VLP productivity under laboratory conditions, their scalability to industrial manufacturing remains uncertain and is likely to present multiple practical and regulatory challenges. Achieving uniform inducer distribution across large bioreactor volumes may be difficult, potentially resulting in heterogeneous expression levels. The financial and regulatory implications of incorporating exogenous inducers, together with the requirement to verify their complete removal from the final product, could further complicate process development. Moreover, the long-term stability and fidelity of cellular responses to induction remain to be established under extended culture conditions. Considering the above, constitutive UGCG overexpression cell lines may offer a more stable and predictable expression profile, obviating the need for external inducers and thereby potentially providing a more practical and robust platform for routine viral and non-viral vector production.

Moreover, the constitutive UGCG-overexpressing cell lines could harbour a promising platform for the production of small EVs and EV-derived biologics, including exosome-associated AAVs (exo-AAV). Through precise modulation of glycosphingolipid metabolism, these cells have the potential to significantly enhance the formation of small extracellular vesicles, which play a crucial role in packaging and efficiently delivering exo-AAV vectors (Issa et al., 2023; György et al., 2015; Schiller et al., 2018). Given the distinct cellular interactions required by various AAV serotypes, further exploration of UGCG-engineered cell lines to produce different exo-AAV serotypes presents exciting avenues for optimizing therapeutic production. This approach holds promise for advancing diverse AAV-based therapies and significantly improving biologics manufacturing processes.

## Materials and methods

### Plasmids and plasmid construction

The sgRNA targeting the AAVS1 locus and the LP donor plasmids were kindly provided by the Department of Biological Sciences, KAIST, Republic of Korea, and were constructed as described by Shin et al. (2020). The LP donor plasmid contained 5′ and 3′ homology arms targeting the AAVS1 locus, a puromycin resistance gene, loxP, EF1α promoter, mCherry-coding sequence, lox2272, and a BGH poly(A) tail. The GFP-2A-Cas9 plasmid was generated previously (Grav et al., 2018). RMCE donor plasmids were generated using the NEBuilder cloning method. The RMCE plasmids used for the generation of a constitutive UGCG-overexpressing cell line contained 4xNFκB-5'/3′ sequences, loxP, synthetic promoters (10, 40, and 100 RPU), UGCG + DYK tag sequences, and lox2272. The NFκB sequence was obtained from Shin et al. (2021), the synthetic promoter sequences from Brown et al. (2017), and the UGCG-coding plasmid was purchased from GenScript (UGCG_OHu61224D_pcDNA3.1+/C-(K)-DYK U192YQPNG0-1). The RMCE plasmid used to generate the inducible system contained NFκB-5'/3′ sequences, loxP, UGCG-coding sequences, poly(A) tail, CMV promoter, Tet-On 3G coding sequences, and lox2272. The Tet-On 3G inducible expression system was purchased from Takara Bio (C631168, Kusatsu, Japan). The plasmid used for VLP production encodes the HIV-Gag protein fused in-frame with enhanced green fluorescent protein (eGFP) (referred to as Gag::eGFP). This plasmid was obtained from Addgene (plasmid no. 80605, Massachusetts, USA). The sequences of all relevant genes and the primers used for cloning are provided in Supplementary Tables S2, S3.

### NEBuilder cloning

All PCR reactions for NEBuilder cloning were carried out using Phusion High-Fidelity DNA Polymerase master mix (M0530S, New England Biolabs, Massachusetts, United States). PCR cycling conditions included an initial denaturation step at 98 °C for 30 s, followed by 30 cycles of denaturation at 98 °C for 10 s, annealing at 65 °C–67 °C for 10 s to 3 min (adjusted based on fragment length), and extension at 72 °C for 30 s. A final extension step was performed at 72 °C for 10 min. PCR products were subsequently analyzed by electrophoresis on a 1% agarose gel.

### Cell culture

The cell line used in this study was a serum-free, suspension-adapted human embryonic kidney HEK293 (HEK293SF-3F6) cell line, obtained from the National Research Council of Canada (Montreal, Canada). Cells were routinely cultured in disposable 125-mL baffled polycarbonate Erlenmeyer flasks with caps (431403, Corning Life Sciences, New York, United States). Cultures were maintained in 25 mL of HyCell TransFx-H medium (SH30939.02, Cytiva Life Sciences, Massachusetts, United States), supplemented with 4 mM GlutaMAX (35050061, Gibco, Thermo Fisher, Massachusett, United States) and 0.1% Pluronic F-68 non-ionic surfactant (24040032, Gibco, Life Technologies, Massachusetts, United States). Cultures were maintained in a HeraCell 150 incubator (Thermo Fisher Scientific, Massachusetts, United States) at 37 °C, 5% CO_2_, and 70% relative humidity, with continuous agitation at 130 rpm provided by a Celltron orbital shaker (Infors HT, Switzerland). Viable cell density (VCD) and cell viability were routinely monitored using a NucleoCounter NC-250 automated cell analyzer (ChemoMetec, Allerød, Denmark).

### Cell line generation

HEK293SF-3F6 cells were transfected at a density of 2 × 10^6^ cells/mL with LP donor, AAVS1-gRNA, and GFP-2A-Cas9 plasmids at a 1:1:1 (w:w:w) ratio using PEIpro transfection reagent (Polyplus-transfection, Illkirch-Graffenstaden, France). Briefly, plasmid DNA was diluted in supplemented HyCell TransFx-H medium and vortexed for 10 s. PEIpro was then added at a DNA:PEI ratio of 1:2 (w/w), vortexed three times for 3 seconds each, and the mixture was incubated for 15 min at room temperature before being added to the cell culture. Three days after transfection, cells were subjected to puromycin (A1113803, Thermo Fisher Scientific, Massachusetts, USA) selection. The optimal concentration of puromycin (2 μg/mL) was determined by testing a range of concentrations on wild-type HEK293SF-3F6 cells (Supplementary Figure S10A). Cells under puromycin selection were passaged every 2–3 days and fully recovered after 22 days (Supplementary Figure S2A). Once cell viability exceeded 97%, the population was subjected to single-cell sorting using fluorescence-activated cell sorting (FACS) with a Sony SH800S cell sorter using a 130 µm sorting chip (Sony Biotechnology Inc., San Jose, United States). mCherry-positive cells, representing successfully integrated populations, were isolated from non-integrated cells. Single cells were seeded into flat-bottom 384-well plates (CLS3821BC, Sigma-Aldrich, St. Louis, United States) in 30 µL of HyCell TransFx-H medium supplemented with 4 mM GlutaMAX, 0.1% Pluronic F-68 non-ionic surfactant, with 5% (V/V) InstiGRO HEK (Advanced Instruments, Norwood, United States), 1.5% HEPES (15630056, Gibco, Thermo Fisher, Massachusetts, United States), and 1% antibiotic-antimycotic (1157048, Gibco, Thermo Fisher, Massachusetts, United States). Fourteen days after single-cell sorting, plates were imaged using a Celigo imaging cytometer (Nexcelom Bioscience, Massachusetts, United States), and the entire volume of subconfluent clones was transferred to 180 µL of HyCell TransFx-H medium supplemented with 4 mM GlutaMAX and 1% Pluronic F-68 non-ionic surfactant in flat-bottom 96-well plates using an epMotion 5,070 liquid handling workstation (Eppendorf, Hamburg, Germany). Verified clones were subsequently expanded in suspension culture in 12-well plates, followed by 6-well plates and finally shake flasks.

To generate stable UGCG cells, AAVS1-LP master cell lines were transfected at a density of 2 × 10^6^ cells/mL using RMCE donor plasmids (10, 40,100 RPU-UGCG and Tet-On-UGCG) together with a Cre-recombinase expression plasmid at a ratio of 3:1 (w:w), employing the PEIpro transfection reagent. At 72 h post-transfection, cells underwent FACS to isolate mCherry-negative cells, indicating successful recombination, from the mCherry-positive population. Sorted single cells were seeded into 384-well plates containing 30 µL of HyCell TransFx-H medium supplemented with 5% (V/V) InstiGRO HEK (Advanced Instruments, Norwood, United States) 4 mM GlutaMAX, 0.1% Pluronic F-68 non-ionic surfactant, 1.5% HEPES, and 1% antibiotic-antimycotic solution. After 14 days, growing clones were transferred into 96-well plates with 180 µL of fresh HyCell TransFx-H medium supplemented with 4 mM GlutaMAX, utilizing an epMotion 5,070 automated liquid handling workstation. Verified clones were subsequently expanded through suspension cultures sequentially in 12-well plates, 6-well plates, and ultimately shake flasks for further downstream analysis.

### Stability of AAVS1 master cell lines

Four final clones with a single integrated copy of the landing pad (LP; lox-mCherry) at the AAVS1 locus were selected for long-term stability analysis. Cells were passaged continuously for 8 weeks in disposable 125-mL baffled shake flasks containing 25 mL culture medium. At each passage, cells were seeded at a density of 0.3 × 10^6^ cells/mL. Stability of cassette integration and mCherry expression levels were assessed regularly by FACS (Supplementary Figures S3D, 11).

### Batch cultures and metabolite profiling

Cells were seeded at a density of 3 × 10^5^ cells/mL in 125 mL baffled Erlenmeyer flasks containing 30 mL of culture medium. Cultures were incubated at 37 °C, with 5% CO_2_ and 70% relative humidity, under continuous shaking at 130 rpm for 10 days. Daily samples were collected to assess VCD, cell viability, percentage of aggregated cells and cell diameter using the NucleoCounter NC-250. For metabolite analysis, 0.5 mL of culture was centrifuged at 1,000 × g for 5 min, and the supernatants were analyzed for glucose, glutamine, glutamate, ammonia, lactate, potassium, and sodium using the BioProfile FLEX2 analyzer (Nova Biomedical, Waltham, MA, United States).

### Junction PCR of targeted regions

To verify the genomic integration of the inserts in both AAVS1 master cell lines and UGCG-overexpressing cell lines, junction PCR analysis (5′ and 3′) was performed. PCR reactions were conducted using OneTaq Quick-Load 2X Master Mix with Standard Buffer (M0486L, New England Biolabs, USA). PCR cycling conditions included an initial denaturation step at 94 °C for 30 s, followed by touchdown cycles comprising denaturation at 94 °C for 30 s, annealing at 68 °C for 60 s, and extension at 68 °C for 1–2 min, with a final extension step at 68 °C for 5 min. The resulting PCR products were analyzed by electrophoresis on a 1% agarose gel. Genomic DNA used in junction PCR assays was extracted from cell pellets harvested from 96-well plates using QuickExtract DNA Extraction Solution (Lucigen, USA), according to the manufacturer’s instructions.

### Copy number analysis by digital PCR

Copy number analysis was performed using QuantStudio Absolute Q Digital PCR (Thermo Fisher Scientific, United States). Briefly, genomic DNA (gDNA) was isolated using the GeneJET Genomic DNA Purification Kit (K0721, Thermo Fisher Scientific, United States) and diluted to 10 ng/μL. Subsequently, diluted gDNA was mixed with Absolute Q DNA Digital Master Mix (A52490, Thermo Fisher Scientific, United States) along with endogenous and transgene-specific TaqMan assays. After thorough mixing, 9 µL of this reaction mixture was loaded into microfluidic array plates (A52865, Thermo Fisher Scientific, United States), followed by the addition of 15 µL of isolation buffer (A52730, Thermo Fisher Scientific, United States). PCR conditions included an initial denaturation at 96 °C for 10 min, followed by 40 cycles of denaturation at 96 °C for 5 s and annealing/extension at 61 °C for 30 s. Copy number was calculated using QuantStudio Absolute Digital PCR Software version 6.3.0. Custom-designed and commercial TaqMan probes were obtained from Thermo Fisher Scientific, with all probes listed in Supplementary Table S2.

### Quantification of UGCG expression by DYKDDDDK-tag ELISA

Quantification of transiently and stably expressed UGCG-DYKDDDDK was performed using the DYKDDDDK-Tag Protein ELISA Kit (AKR-5188, Cell Biolabs Inc., California, United States). For transient expression, HEK293SF-3F6 cells were transfected at a density of 2 × 10^6^ cells/mL with Gag::eGFP and UGCG-DYKDDDDK plasmids at a ratio of 1:1 (w:w) using PEIpro transfection reagent. Three days post-transfection, transfection efficiency was assessed, and 1 × 10^7^ cells were harvested for UGCG-DYKDDDDK quantification. Similarly, 1 × 10^7^ cells were harvested from UGCG-DYKDDDDK overexpressing stable cell lines for analysis. Harvested cells were lysed by performing three freeze-thaw cycles and subsequently resuspended in TMS lysis buffer (50 mM Tris-Cl, 150 mM NaCl, 2 mM MgCl_2_, pH 8.0 adjusted with NaOH). Cell lysates and recombinant DYKDDDDK-tagged GST protein standards were added to a DYKDDDDK-conjugate-coated microplate. Following a brief incubation, anti-DYKDDDDK monoclonal antibody was added. After five washes with wash buffer, an HRP-conjugated secondary antibody was introduced. The plate was again washed and substrate solution added, inducing a colorimetric reaction. The reaction was stopped after sufficient color development, and absorbance was measured at 450 nm using an Epoch 2 Microplate Reader (BioTek, Vermont, United States). The concentration of DYKDDDDK-tagged protein in samples was calculated by comparison to standard curve generated from recombinant standards. Amino acid sequances of UGCG-DYKDDDDK is avaiable in Supplementary Table S3.

### Quantification of VLPs and EVs by nanoparticle tracking analysis (NTA)

Extracellular vesicles and Gag::eGFP VLPs were quantified using NTA. Measurements were performed on a NanoSight NS300 instrument (NanoSight Ltd, Malvern, UK) equipped with a blue laser module (488 nm) for fluorescence-based detection of Gag::eGFP VLPs, and a neutral-density filter for total particle detection via light scattering. Data analysis was carried out using NanoSight NTA software version 3.1. Two types of analysis were carried out: total diffracting particles (EVs + VLPs) and fluorescent particles (only VLPs).

### Flow cytometry and flow virometry

The percentage of cells positive for Gag::eGFP was determined using a CytoFLEX flow cytometer (Beckman Coulter, United States). Gag::eGFP VLPs were also quantified using the CytoFLEX instrument. Instrument settings included forward scatter (FSC) gain at 70, side scatter (SSC) gain at 70, violet side scatter (V-SSC) gain at 9, and FITC fluorescence (525/40 nm) gain at 500. Prior to analysis, samples were diluted in filtered phosphate-buffered saline (11594516, ThermoFisher Sintific, United States) to obtain a particle concentration between 500 and 5,000 events/µL, maintaining an abort rate below 5%. A minimum of 20,000 events representing Gag::eGFP VLPs were recorded per sample. Data acquisition was performed at a low flow rate (10 μL/min), and VLPs were identified based on V-SSC and FITC fluorescence signals using V-SSC as the triggering parameter. Data analysis was conducted using CytExpert software (version 2.3, Beckman Coulter, United States). VLP concentration were calculated using Equation 1 and nomalised with a standard (S_NTA_) to harmonize measurements between NTA and CytoFLEX methods.

$$VLPConc.\left(\frac{VLPs}{mL}\right)=\frac{events}{\mu l}\cdot \frac{1000\mu l}{mL}\cdot Dilution\cdot S_{NTA}$$ (1)

### Optimization of inducible system by design of experiments (DOE)

A Rotatable Central Composite Design (RCCD) was employed to investigate the effect of UGCG gene activation using the Tet-On system on VLP specific productivity and transfection efficiency. The two independent variables evaluated were doxycycline concentration (ng/mL) and induction time (hours post-transfection). The RCCD included 4 factorial points, 4 axial points (α = ±√2), and 5 center points, resulting in a total of 13 experimental runs. Factor levels were coded as −1 (low), 0 (center), and +1 (high). Experimental data were fitted to a second-order polynomial model described by Equation 2.

$$y=\beta_{0}+\sum \beta_{i}x_{i}+\sum \beta_{ii}{x_{i}}^{2}+\sum \beta_{ij}x_{i}x_{j}+\varepsilon$$ (2)

where *y* represents the response variable (VLP specific productivity or transfection efficiency), *β*_*0*_ is the intercept, *βᵢ* the linear coefficient, *βᵢᵢ* the quadratic coefficient, *β_ij_* the interaction coefficient, *Xᵢ* and *X_j_* are the independent variables, and ε is the experimental error. Model generation, analysis, and prediction of optimal conditions were performed using JMP^®^ Pro 17 (Version 17.0.0, SAS Institute Inc., United States).

### Ceramide supplementation

Two different conditions were tested. The cell lines were passaged and cultured in media supplemented with ceramide or ceramide was added immediately before transfection. The ceramide stock was prepared dissolving C2 Ceramide (860502P, Sigma Aldrich) in dimethyl sulfoxide (DMSO) at a concentration of 1 mg/mL. After this, the corresponding volume was added to the culture media to achieve a final concentration of 2 μg/mL, for both supplementation strategies.

### Multiplexed quantitative proteomic analysis

Cells were grown to 2 × 10^6^ cells/mL and 1.5 mL of culture was centrifuged at 300g for 10 min. After discarding the supernatant, the pellet was stored at −70 °C for proteomics. Protein identification was performed as described previously (Catalán-Tatjer et al., 2025). In this case, TMT-16plex was used. Briefly, MS/MS scans were matched against a *Homo sapiens* together with Adenovirus 5 (Ad5) (UniProtKB/Swiss-Prot 2023–10 Release). For comparative analysis of changes in protein abundance, we applied weighted scan−peptide−protein statistical workflow, using SanXoT package (Navarro et al., 2014; Martínez-Acedo et al., 2012). The quantitative information is, as detailed by Lavado-García et al. (Lavado-García et al., 2020), obtained from the spectra and used to quantify the peptides from which the spectra are produced and then the proteins that generate these peptides. These standardized variables (Zq) express the quantitative values in units of standard deviation (Navarro et al., 2014). The quantified proteins were functionally annotated using the Gene Ontology (GO) database (Ashburner et al., 2000). For further Gene Ontology annotation, the database for annotation, visualization and integrated discovery (DAVID) was used to perform functional enrichment analysis and to extract adjusted Benjamini-Hochberg scores for the enriched processes (Benjamini and Hochberg, 1995; Huang et al., 2009).

### Data management and statistical analysis

Graphs were generated using GraphPad Prism v.10.1.0. Statistical analysis was performed using the same software, employing one-way ANOVA and Dunnett’s test with a 95% confidence interval. All experiments were conducted with three biological replicates.

## Data Availability

The datasets presented in this study can be found in online repositories. The names of the repository/repositories and accession number(s) can be found in the article/Supplementary Material.
