# Supplementary material for: Targeted UDP-glucose ceramide glucosyltransferase stable overexpression induces a metabolic switch improving cell performance at high cell density
Source: Front Bioeng Biotechnol. 2025 Oct 13;13:1690203. doi: 10.3389/fbioe.2025.1690203 (PMC12556263; doi:10.3389/fbioe.2025.1690203)

Supplementary Figure S1: Plasmid Maps: These plasmids were used for the generation of HEK-SF-3F6-AAVS1-LP master cell line (MCL): (A) GFP-2A-Cas9, (B) AAVS1-LP donor, and (C) AAVS1-gRNA.

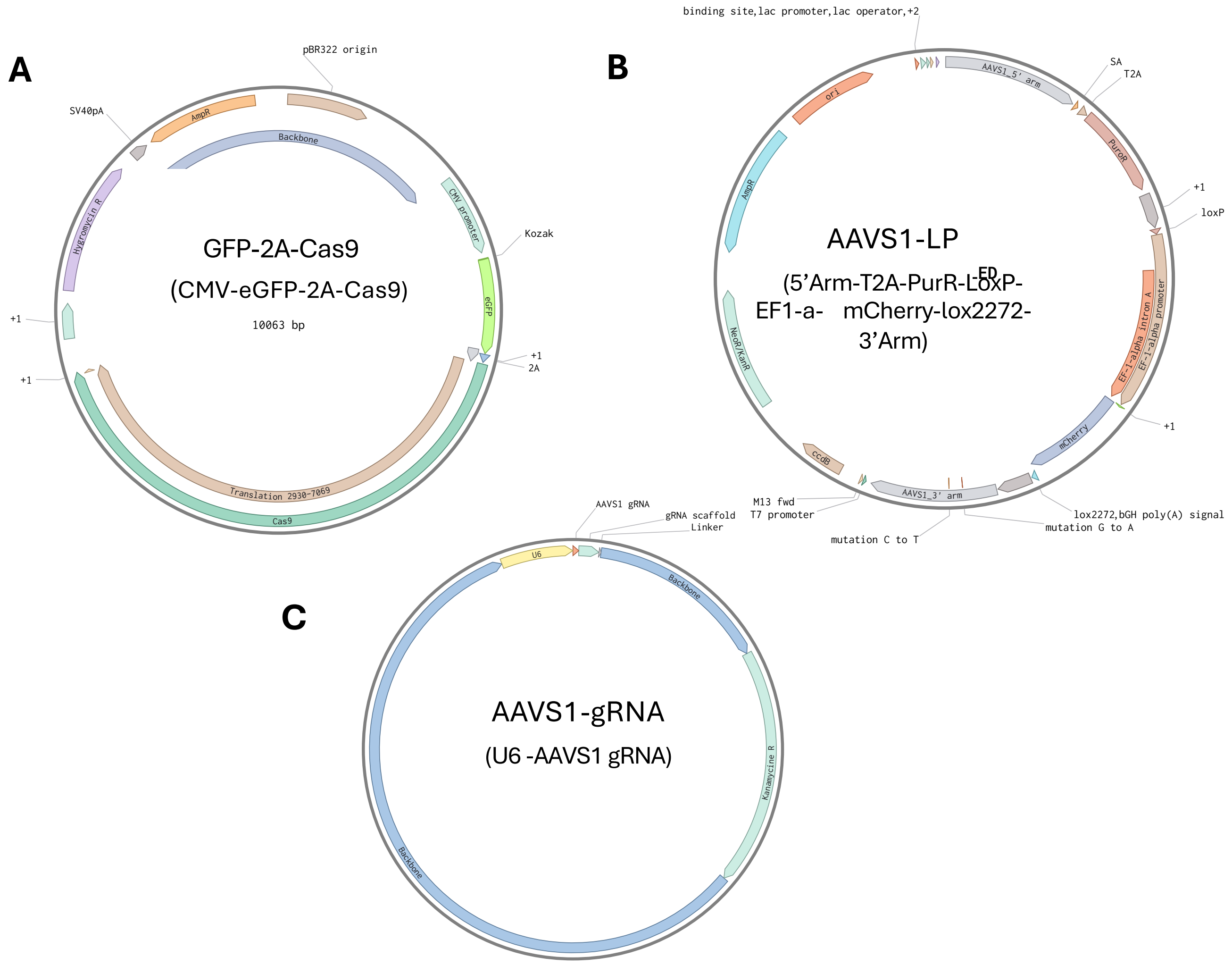

Supplementary Figure S2: (A) HEK-SF-3F6-AAVS1-LP MCL during puromycin selection. (B) The percentage of mCherry positive population after antibiotic selection during single cell sorting. the marked area (52.21%) was sued for sorting. LP=landing pad.

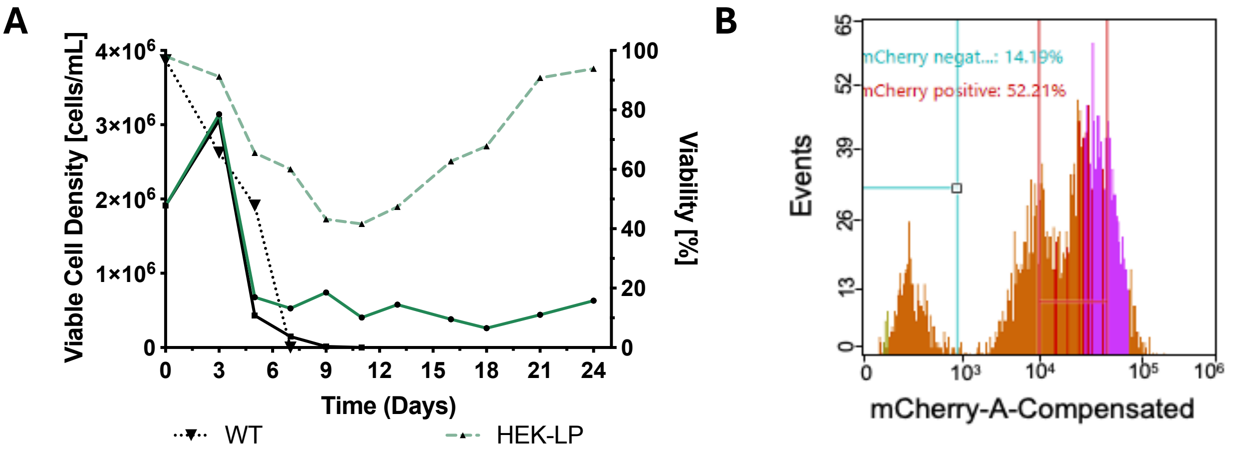

Supplementary Figure S3: Junction PCR gel image on 1% agarose. (A) AAVS1-LP 5' junction PCR, expected band 1500 bps . (B) AAVS1-LP 3' junction PCR, expected band 500 bps. (C) mCherry copy number was quantified relative to the endogenous COSMC gene. (D) Long-term (8 weeks) stability analysis of three selected clones, evaluating viable cell density (VCD) and viability.

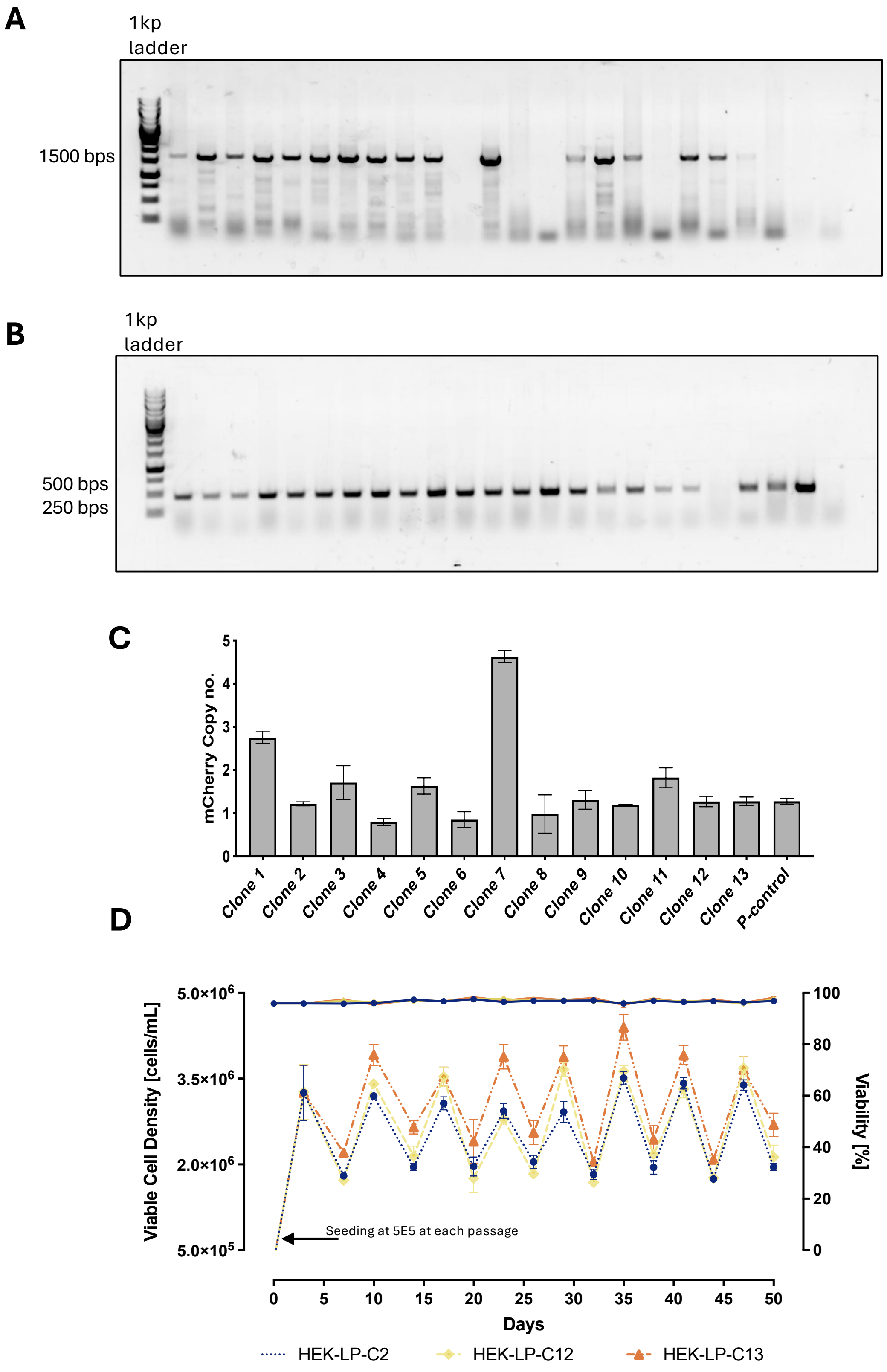

Supplementary Figure S4: Plasmid map: Plasmids used for generation of UGCG overexpressing constitutive cell lines : (A) UGCG-10 RPU, (B) UGCG 40 RPU, (C) UGCG 100 RPU and (D) Tet-On system.

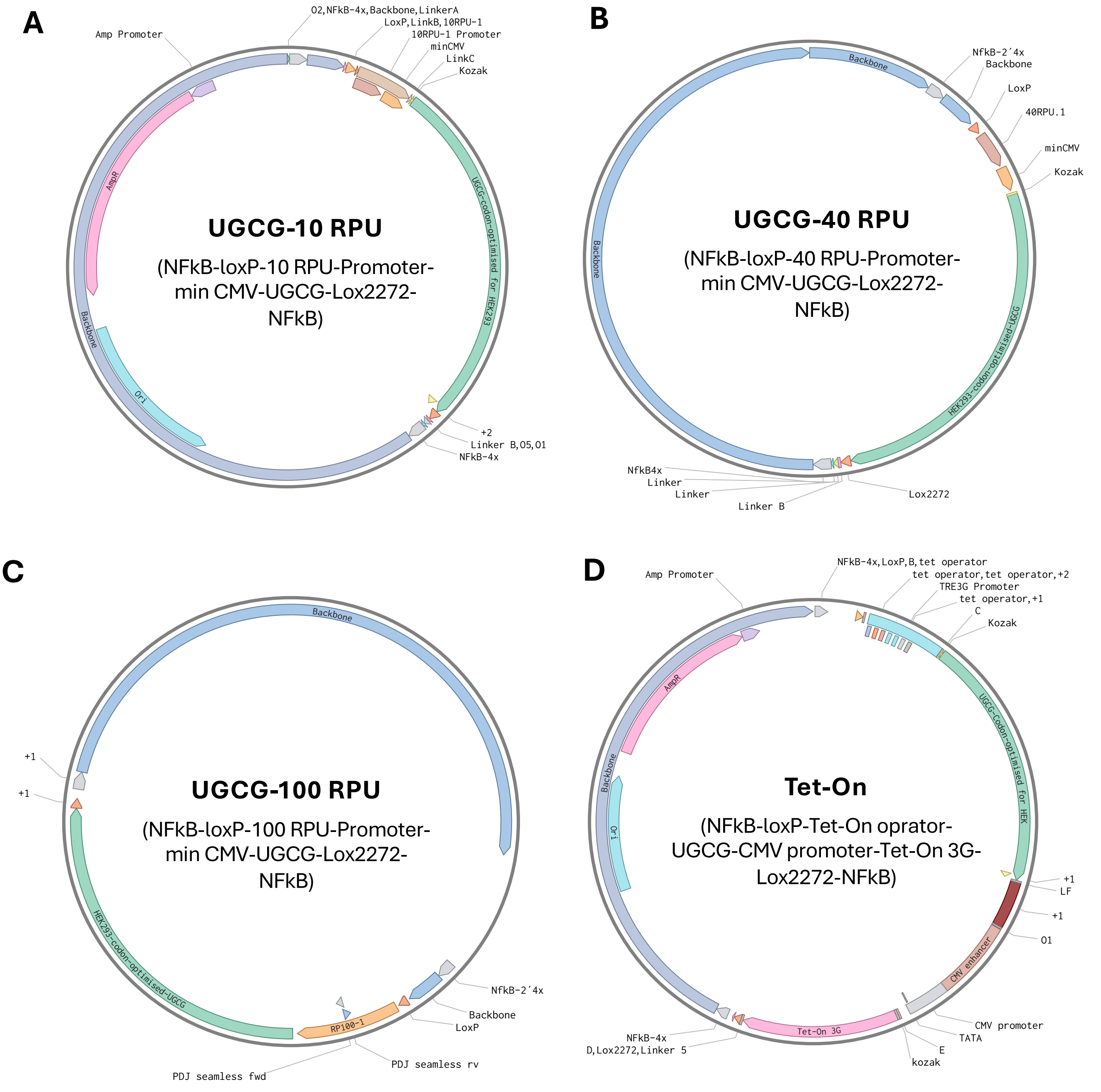

Supplementary Figure S5: Representative FACS plots and gating strategy for RMCE in stable cell lines. (A) Percentage of mCherry-negative cells, indicating successful cassette exchange via RMCE when NFκB is present in the RMCE donor plasmids. (B) Percentage of mCherry-negative cells, indicating cassette exchange in the absence of NFκB in the RMCE donor plasmids.

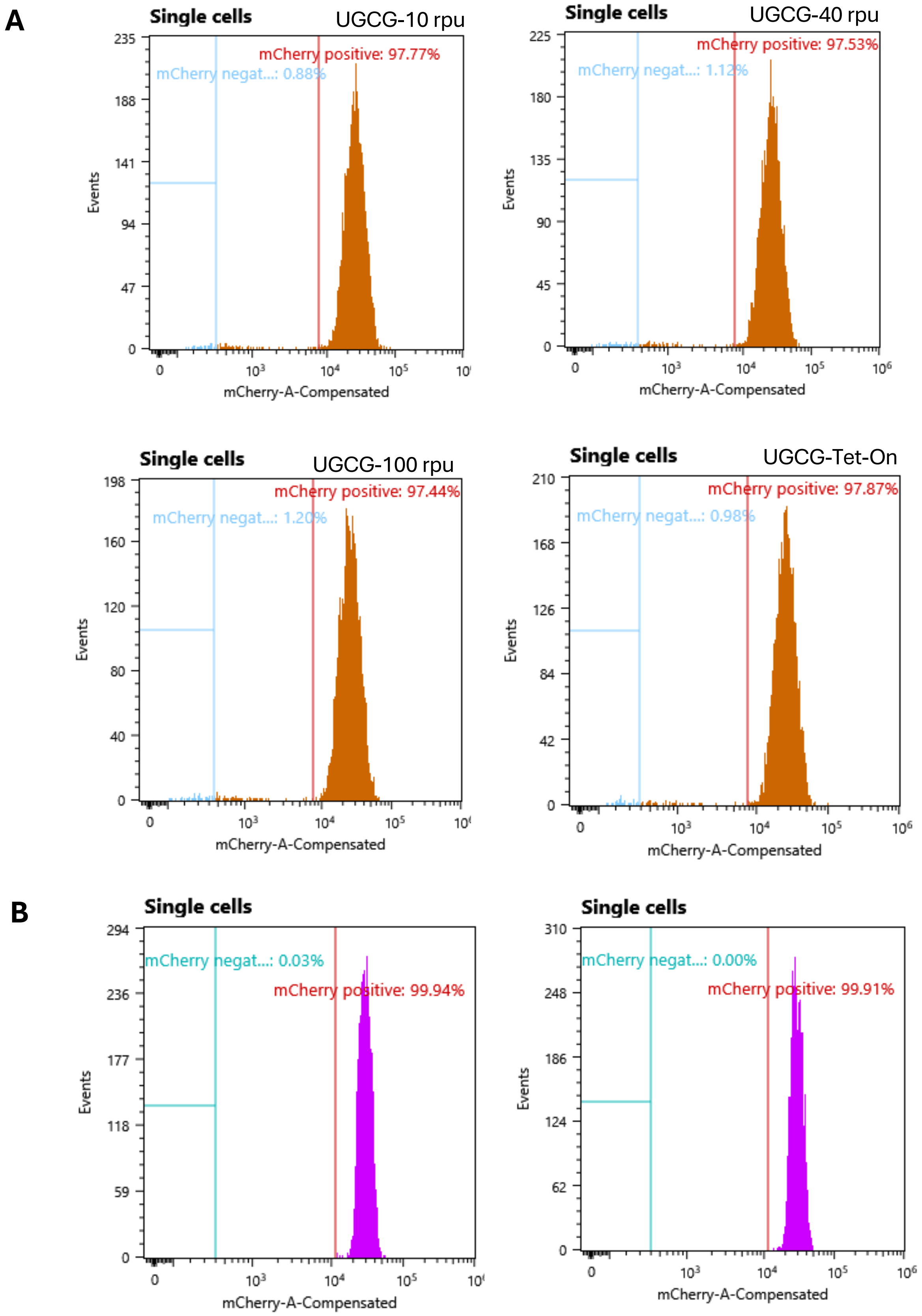

Supplementary Figure S6: Junction PCR gel image on 1% agarose. A (3'-UGCG-10 RPU), B (3'-UGCG-40 RPU), C (3'-UGCG-100 RPU), D (3'-UGCG-Tet-On). E (5'-UGCG-10 RPU), F (5'-UGCG-40 RPU), G (5'-UGCG-100 RPU), H (5'-UGCG-Tet-On).

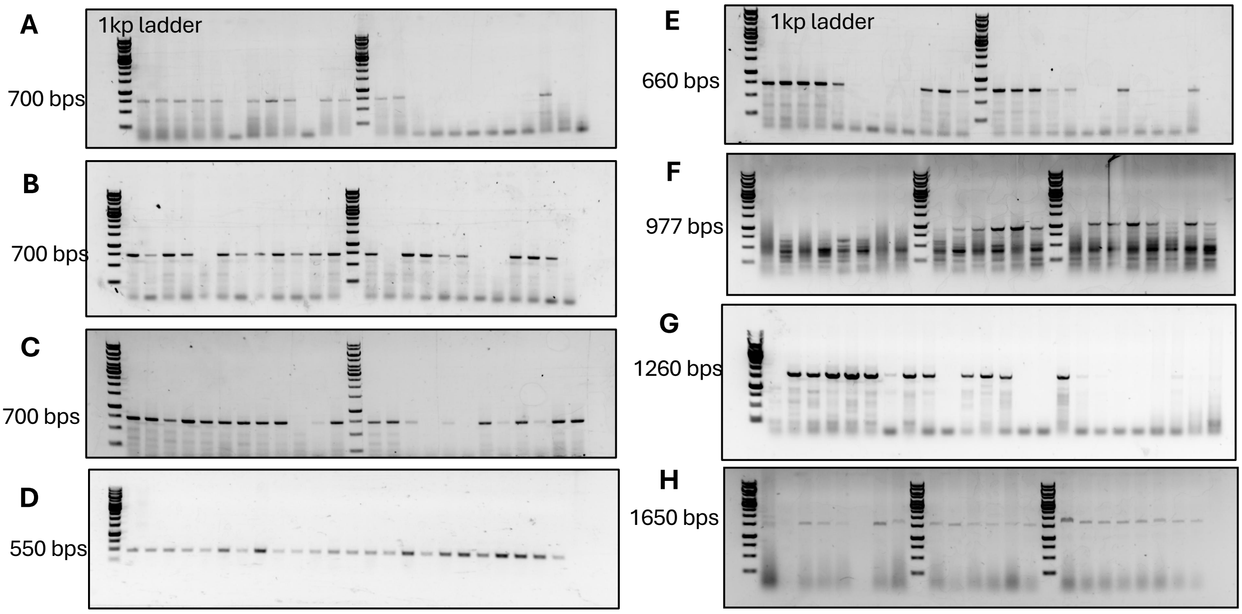

Supplementary Figure S7: Copy Number Analysis of UGCG & Tet-On in stable overexpressing cell lines. UGCG copy number was quantified relative to the endogenous reference gene UBC. The original UGCG copies present in the parental HEK cells were subtracted to determine the number of integrated UGCG copies. (A) UGCG copies in 10 RPU clones (B) UGCG copies in 40 RPU clones (C) UGCG copies in 100 RPU clones, (D) UGCG copies in inducible clones (E) Tet-On copies in inducible clones. Clones with one copy of targeted were selected for downstream experiments.

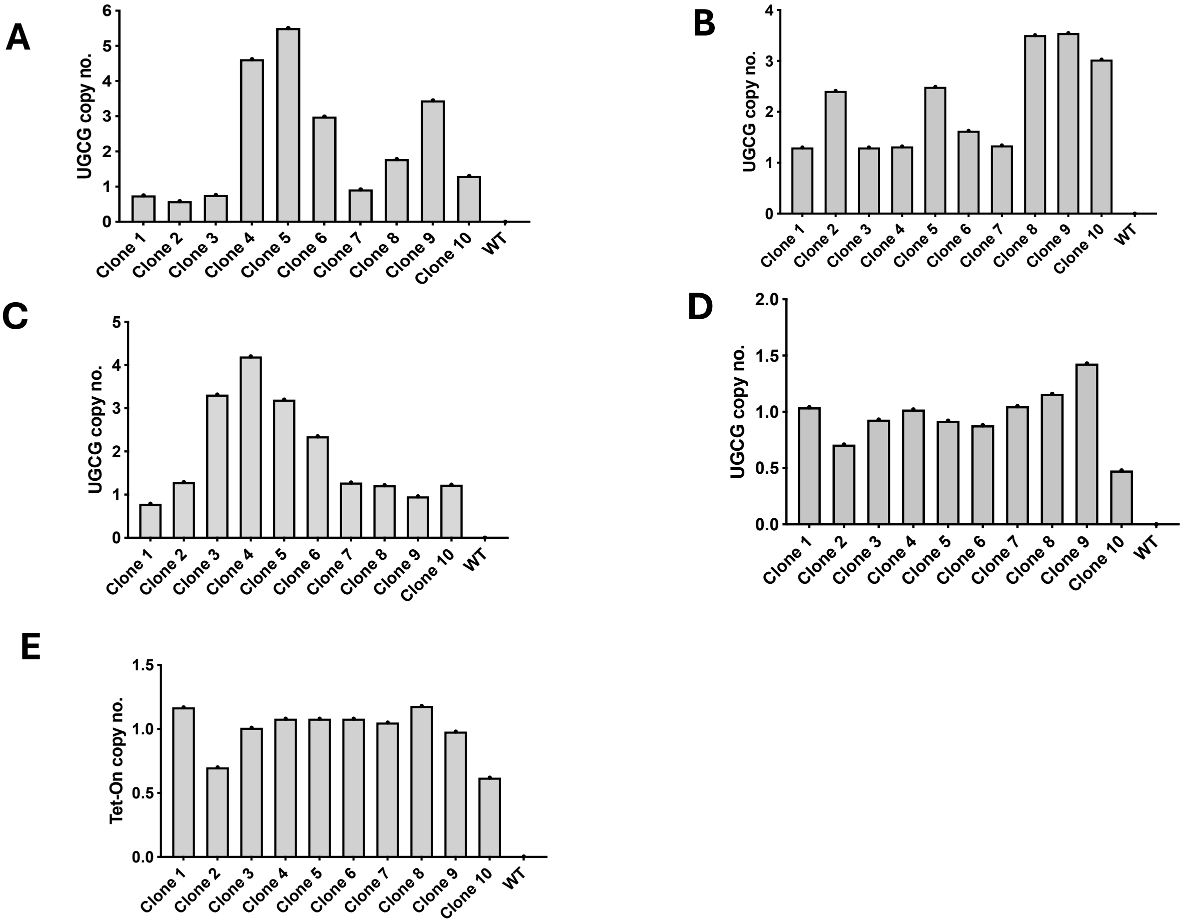

Supplementary Figure S8. Comparison of cell growth, viability, transfection efficiency, and specific productivity under two conditions: ceramide addition at the time of transfection (A–D at 2, 3, 8 and 10 M cells/mL) and continuous culture in ceramide (E-H at 2M cells/mL). (A, E) Viable cell density (VCD, cells/mL), (B, F) cell viability (%), (C, G) GFP-positive cell percentage (%), and (D, H) specific productivity (qp, particles/cell/day). All experiments were performed in three biological triplicates. Statistical significance was determined using one-way ANOVA and Dunnetts' test: ****p < 0.0001, ***p < 0.001, **p < 0.01, *p < 0.05. M=million cells.

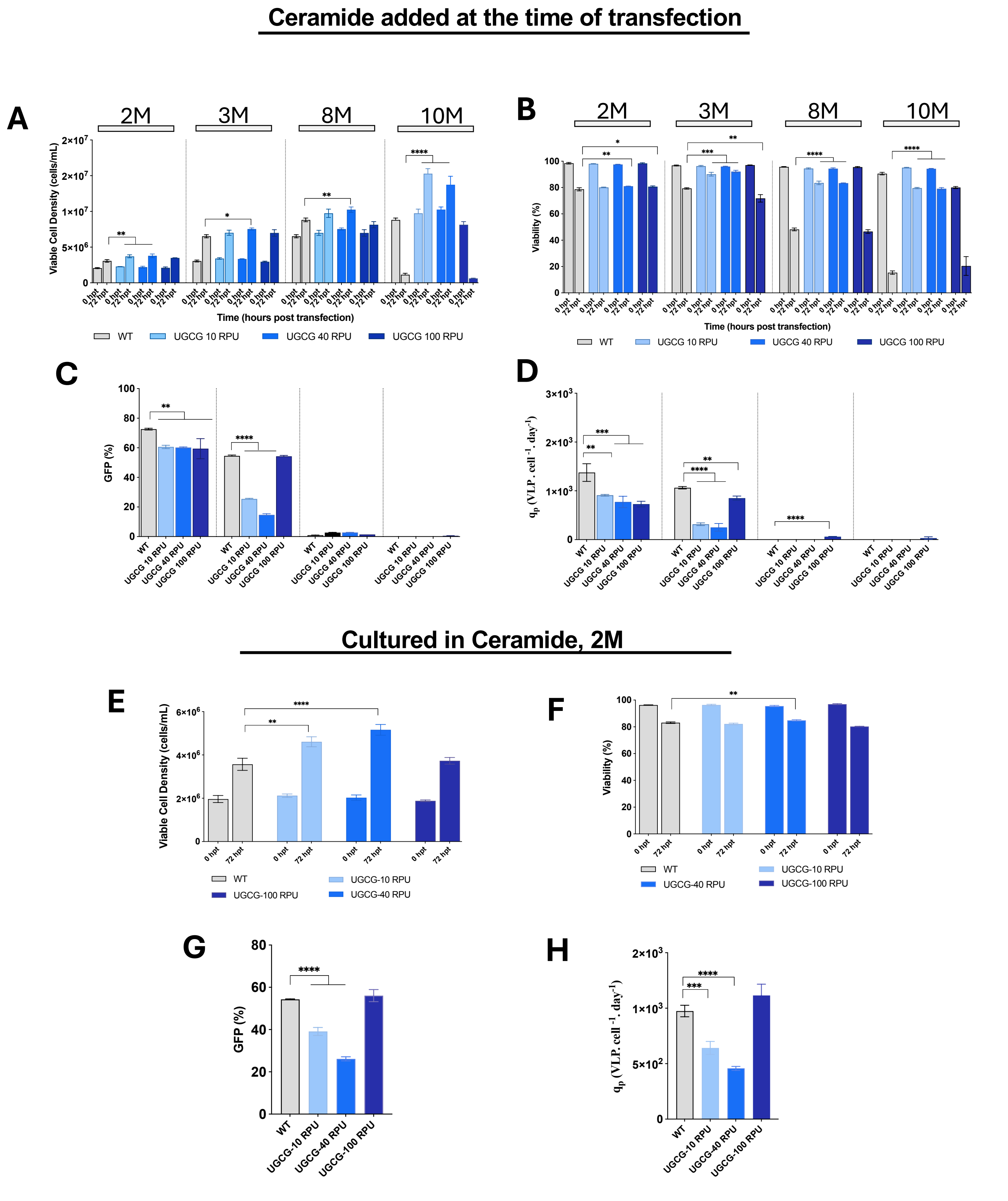

Supplementary Figure S9. (A) Three-dimensional response surface plot demonstrating the combined effect of inducer concentration and induction time on transfection efficiency, measured as GFP (%) and VLP specific productivity. (B) Prediction Profiler showing the modeled relationship between inducer concentration, induction time, transfection efficiency, and VLP productivity. (C) Transfection efficiency represented by the percentage of GFP-positive cells. (D) Specific productivity of virus-like particles (VLPs). Cell cultures were induced with 81.78 ng/µL doxycycline at 5.5 hours post-transfection (hpt). All experiments were performed in three biological triplicates. Statistical significance was determined using one-way ANOVA and Dunnett's test: $^{**}p < 0.01$, $^{*}p < 0.05$. (E) Correlation plots showing the relationship between promoter strength and transfection efficiency (GFP%), as well as extracellular vesicle (EV) and virus-like particle (VLP) production.

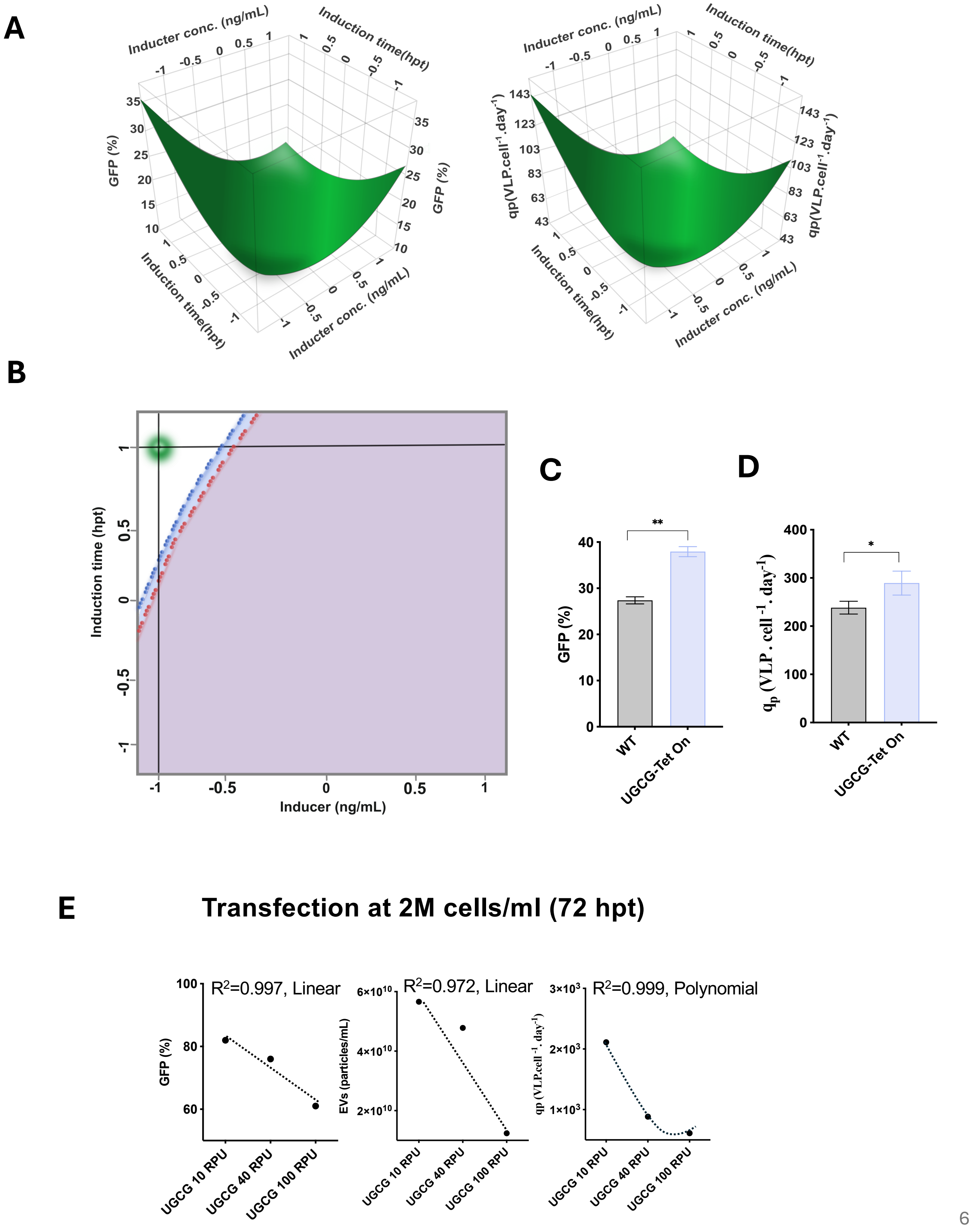

Supplementary Table S2: Sequence information.

GOI	Gene sequances (5’-> 3’)
**AAVS1-5’ Arm**	CCGTCTCTCTCCTGAGTCCGGACCACTTTGAGCTCTACTGGCTTCTGCGCCGCCTCTGGCCCACTGTTTCCCCTTCCCAGGCAGGTC<br>CTGCTTTCTCTGACCTGCATTCTCTCCCCTGGGCCTGTGCCGCTTTCTGTCTGCAGCTTGTGGCCTGGGTCACCTCTACGGCTGGCCC<br>AGATCCTTCCCTGCCGCCTCCTTCAGGTTCCGTCTTCCTCCACTCCCTCTTCCCCTTGCTCTCTGCTGTGTTGCTGCCCAAGGATGCT<br>CTTTCCGGAGCACTTCCTTCTCGGCGCTGCACCACGTGATGTCCTCTGAGCGGATCCTCCCCGTGTCTGGGTCCTCTCCGGGCATCT<br>CTCCTCCCTCACCCAACCCCATGCCGTCTTCACTCGCTGGGTTCCCTTTTCCTTCTCCTTCTGGGGCCTGTGCCATCTCTCGTTTCTTA<br>GGATGGCCTTCTCCGACGGATGTCTCCCTTGCGTCCCGCCTCCCCTTCTTGTAGGCCTGCATCATCACCGTTTTTCTGGACAACCCCA<br>AAGTACCCCGTCTCCCTGGCTTTAGCCACCTCTCCATCCTCTTGCTTTCTTTGCCTGGACACCCCGTTCTCCTGTGGATTCGGGTCAC<br>CTCTCACTCCTTTCATTTGGGCAGCTCCCCTACCCCCCTTACCTCTCTAGTCTGTGCTAGCTCTTCCAGCCCCCTGTCATGGCATCTTC<br>CAGGGGTCCGAGAGCTCAGCTAGTCTTCTTCCTCCAACCCGGGCCCCTATGTCCACTTCAGGACAGCATGTTTGCTGCCTCCAGGGA<br>TCCTGTGTCCCCGAGCTGGGACCACCTTATATTCCCAGGGCCGGTTAATGTGGCTCTGGTTCTGGGTACTTTTATCTGTCCCCTCCAC<br>CCCACAGT
**AAVS1-3’Arm**	GGGGCCACTAGGGACAGGATTGGTGACAGAAAAGCCCCATCCTTAGGCCTCCTCCTTCCTAGTCTCCTGATATTGGGTCTAACCCCC<br>ACCTCCTGTTAGGCAGATTCCTTATCTGGTGACACACCCCCATTTCCTGGAGCCATCTCTCTCCTTGCCAGAACCTCTAAGGTTTGCTT<br>ACGATGGAGCCAGAGAGGATCCTGGGAGGGAGAGCTTGGCAGGGGGTGGGAGGGAAGGGGGGGATGCGTGACCTGCCCGGTTCT<br>CAGTGGCCACCCTGCGCTACCCTCTCCCAGAACCTGAGCTGCTCTGACGCGGCCGTCTGGTGCGTTTCACTGATCCTGGTGCTGCA<br>GCTTCCTTACACTTCCCAAGAGGAGAAGCAGTTTGGAAAAACAAAATCAGAATAAGTTGGTCCTGAGTTCTAACTTTGGCTCTTCACCTT<br>TCTAGTCCCCAATTTATATTGTTCCTCCGTGCGTCAGTTTTACCTGTGAGATAAGGCCAGTAGCCAGCCCCGTCCTGGCAGGGCTGTG<br>GTGAGGAGGGGGGTGTCCGTGTGGAAAACTCCCTTTGTGAGAATGGTGCGTCCTAGGTGTTCACCAGGTCGTGGCCGCCTCTACTCC<br>CTTTCTCTTTCTCCATCCTTCTTTCCTTAAAGAGTCCCCAGTGCTATCTGGGACATATTCCTCCGCCCAGAGCAGGGTCCCGCTTCCCT<br>AAGGCCCTGCTCTGGGCTTCTGGGTTTGAGTCCTTGGCAAGCCCAGGAGAGGCGCTCAGGCTTCCCTGTCCCCCTTCCTCGTCCAC<br>CATCTCATGCCCCTGGCTCTCCTGCCCCTTCCCTACAGGGGTTCCT
**AAVS1-gRNA**	ACCCCACAGTGGGGCCACTA

Primers used for junction PCR of AAVS1-LP cell lines.

**Primer name**	**Primer seq 5’->3’**
**5’ –Junction PCR-Fw**	GGACTTTGTCTCCTTCCCTG
**5’ –Junction PCR-Rev**	GGAGGCCTTCCATCTGTTGC
**3’ -Junction PCR-Fw**	TGAGGTCAAGACCACCTACA
**3’ -Junction PCR-Rev**	ATGACGTCTCCATAGAGCCCACCGCATCC

Tag-Man assays used for copy number determination.

GOI	FW Primer (5’->3’)	Rev Primers (5’-> 3’)	Tagman Prop (5’->3’)
**mCherry (FAM-MGB)**	GACTACTTGAA GCTGTCCTTCC	CGCAGCTTCACCT TGTAGAT	TTCAAGTGGGAGCGCGTGATGAA
**Cosmc (VIC-MGB)**	ACCCGAACCAG GTAGTAGAA	ACATGTCCAAAGG CCCTAAG	AGTGACAGCCATATTGGAACAGCATCC
**UGCG (FAM-MGB)**	-	-	Comercial, Assay ID: Hs00916612-m1
**Tet-On (FAM-MGB)**	-	-	Comercial, Assay ID: ARU67PP
**UBC (VIC-MGB)**	-	-	Comercial, Assay ID: Hs05002522-g1

Supplementary Figure S10: (A) Determination of optimal (2ug/mL) antibiotic concentration for selection.

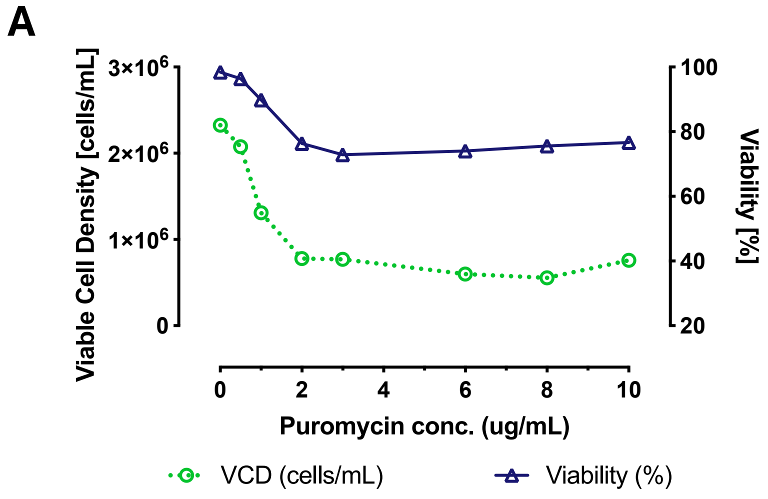

Supplementary Table S3: Sequence information.

GOI	Gene sequances (5'->3')
**10 RPU.1-Promoter**	TGGGGCGGGGAGTATACGACCTATTTTGCGCAATTCTGATAAAGGTCTTACCGGAAGTTCAGATTTATAGGTTTT<br>GCGCAATTAGTATGCCTTAGTGGGGCGGGGA
**40 RPU.1-Promoter**	TTTTGCGCAATTTATAGGTGGGGCGGGGAAAGGTCATGACACAGCAATCAGATTTGCTTGCGTGAGAAGAAGT<br>ATGTTACCGGAAGTTGACCTATGGGACTTTCCATCTAACATGACACAGCAAT
**100 RPU.1-Promoter**	TGGGACTTTCCACCTTAGATGACACAGCAATCAGATTTGCTTGCGTGAGAAGATATAGGATGACACAGCAATCT<br>AGACTGGGACTTTCCACTGATATTTTGCGCAATTGACCTAATGACACAGCAATAGTATGTGGGGCGGGGATCT<br>AACTGGGACTTTCCAAAGGTCTTACCGGAAGTTGTTAGAATGACACAGCAATGGATTCATATCCTGGGACTTTC<br>CAGTATACTGCTTGCGTGAGAAGATGATCATGGGACTTTCCATGTACAAAAGGTCTATATAAGCAGAGCTCGTT<br>TAGTGAACCGTCAGATCGCCTAGATACGCCATCCACGCTGTTTTGACCTCCATAGAAGAC
**NFkB-4x**	CTGGGGACTTTCCAGCTGGGGACTTTCCAGCTGGGGACTTTCCAGCTGGGGACTTTCCAGG
**UGCG**	ATGGCGCTGCTGGACCTGGCCTTGGAGGGAATGGCCGTCTTCGGGTTCGTCCTCTTCTTGGTGCTGTGGCTG<br>ATGCATTTCATGGCTATCATCTACACCCGATTACACCTCAACAAGAAGGCAACTGACAAACAGCCTTATAGCAA<br>GCTCCCAGGTGTCTCTCTTCTGAAACCACTGAAAGGGGTAGATCCTAACTTAATCAACAACCTGGAAACATTCT<br>TTGAATTGGATTATCCCAAATATGAAGTGCTCCTTTGTGTACAAGATCATGATGATCCAGCCATTGATGTATGTAA<br>GAAGCTTCTTGGAAAATATCCAAATGTTGATGCTAGATTGTTTATAGGTGGCAAAAAAGTTGGCATTAATCCTAAA<br>ATTAATAATTTAATGCCAGGATATGAAGTTGCAAAGTATGATCTTATATGGATTTGTGATAGTGGAATAAGAGTAAT<br>TCCAGATACGCTTACTGACATGGTGAATCAAATGACAGAAAAAGTAGGCTTGGTTCACGGGCTGCCTTACGTA<br>GCAGACAGACAGGGCTTTGCTGCCACCTTAGAGCAGGTATATTTTGGAACTTCACATCCAAGATACTATATCTC<br>TGCCAATGTAACTGGTTTCAAATGTGTGACAGGAATGTCTTGTTTAATGAGAAAAGATGTGTTGGATCAAGCAGG<br>AGGACTTATAGCTTTTGCTCAGTACATTGCCGAAGATTACTTTATGGCCAAAGCGATAGCTGACCGAGGTTGGA<br>GGTTTGCAATGTCCACTCAAGTTGCAATGCAAAACTCTGGCTCATATTCAATTTCTCAGTTTCAATCCAGAATGAT<br>CAGGTGGACCAAACTACGAATTAACATGCTTCCTGCTACAATAATTTGTGAGCCAATTTCAGAATGCTTTGTTGC<br>CAGTTTAATTATTGGATGGGCAGCCCACCATGTGTTCAGATGGGATATTATGGTATTTTTCATGTGTCATTGCCTG<br>GCATGGTTTATATTTGACTACATTCAACTCAGGGGTGTCCAGGGTGGCACACTGTGTTTTTCAAAACTTGATTAT<br>GCAGTCGCCTGGTTCATCCGCGAATCCATGACAATATACATTTTTTTGTCTGCATTATGGGACCCAACTATAAG<br>CTGGAGAACTGGTCGCTACAGATTACGCTGTGGGGGTACAGCAGAGGAAATCCTAGATGTAGATTACAAGGA<br>TGACGACGATAAGTGA
**Lox P**	ATAACTTCGTATAGCATACATTATACGAAGTTAT
**Lox2272**	ATAACTTCGTATAGGATACTTTATACGAAGTTAT
**TRE3G Promoter**	TTTACTCCCTATCAGTGATAGAGAACGTATGAAGAGTTTACTCCCTATCAGTGATAGAGAACGTATGCAGACTTT<br>TACTCCCTATCAGTGATAGAGAACGTATAAGGAGTTTACTCCCTATCAGTGATAGAGAACGTATGACCAGTTTA<br>CTCCCTATCAGTGATAGAGAACGTATCTACAGTTTACTCCCTATCAGTGATAGAGAACGTATATCCAGTTTACTC<br>CCTATCAGTGATAGAGAACGTATAAGCTTTAGGCGTGTACGGTGGGCGCCTATAAAAGCAGAGCTCGTTTAGT<br>GAACCGTCAGATCGCCTGGAGCAATTCCACAACACTTTTGTCTTATACCAACTTTCCGTACCACTTCCTACCCT<br>CGTAAA
**Tet On 3G**	ATGTCTAGACTGGACAAGAGCAAAGTCATAAACTCTGCTCTGGAATTACTCAATGGAGTCGGTATCGAAGGCC<br>TGACGACAAGGAAACTCGCTCAAAAGCTGGGAGTTGAGCAGCCTACCCTGTACTGGCACGTGAAGAACAAG<br>CGGGCCCTGCTCGATGCCCTGCCAATCGAGATGCTGGACAGGCATCATACCCACTCCTGCCCCCTGGAAG<br>GCGAGTCATGGCAAGACTTTCTGCGGAACAACGCCAAGTCATACCGCTGTGCTCTCCTCTCACATCGCGAC<br>GGGGCTAAAGTGCATCTCGGCACCCGCCCAACAGAGAAACAGTACGAAACCCTGGAAAATCAGCTCGCGTT<br>CCTGTGTCAGCAAGGCTTCTCCCTGGAGAACGCACTGTACGCTCTGTCCGCCGTGGGCCACTTTACACTGG<br>GCTGCGTATTGGAGGAACAGGAGCATCAAGTAGCAAAAGAGGAAAGAGAGACACCTACCACCGATTCTATG<br>CCCCCACTTCTGAAACAAGCAATTGAGCTGTTCGACCGGCAGGGAGCCGAACCTGCCTTCCTTTTCGGCCT<br>GGAACTAATCATATGTGGCCTGGAGAAACAGCTAAAGTGCGAAAGCGGCGGGCCGACCGACGCCCTTGAC<br>GATTTTGACTTAGACATGCTCCCAGCCGATGCCCTTGACGACTTTGACCTTGATATGCTGCCTGCTGACGCTC<br>TTGACGATTTTGACCTTGACATGCTCCCCGGGTAA
**DYKDDDK-UGCG (AA)**	ATMALLDLALEGMAVFGFVLFLVLWLMHFMAIIYTRLHLNKKATDKQPYSKLPGVSLLKPLKGVDPNLINNLETFFELDYPKYEVLL<br>CVQDHDDPAIDVCKKLLGKYPNVDARLFIGGKKVGINPKINNLMPGYEVAKYDLIWICDSGIRVIPDTLTDMVNQMTEKVGLVH<br>GLPYVADRQGFAATLEQVYFGTSHPRYYISANVTGFKCVTGMSCLMRKDVLDQAGGLIAFAQYIAEDYFMAKAIADRGWRFAMS<br>TQVAMQNSGSYSISQFQSRMIRWTKLRINMLPATIICEPISECFVASLIIGWAAHHVFRWDIMVFFMCHCLAWFIFDYIQLRGVQ<br>GGTLCFSKLDYAVAWFIRESMTIYIFLSALWDPTISWRTGRYRLRCGGTAEEILDVDYKDDDDK--

Supplementary Table S4: Primers used for the generation of UGCG 40 RPU and UGCG 100 RPU Plasmids

Primer name	Primer seq 5'->3'
Backbone-HEK-UGCG_fwd	catagaagacAGACGTCATCGCCACCATG
Insert fragmen-40rpu_fwd	taagcagcgtTTTTGCGCAATTTATAGGTG
Backbone-HEK-UGCG_fwd	catagaagacAGACGTCATCGCCACCATG
Insert-100rpu-promoter_fwd	taagcagcgtTGGGACTTTCCACCTTAG
Backbone-HEK-UGCG-40rpu_rev	ttgcgcaaaaACGCTGCTTATAACTTCGTATAATGTATG
Insert fragmen-40rpu_Rev	gatgacgtctGTCTTCTATGGAGGTCAAAAC
Backbone-HEK-UGCG-100rpu_rev	gaaagtcccaACGCTGCTTATAACTTCGTATAATGTATG
Insert-100rpu-promoter_rev	gatgacgtctGTCTTCTATGGAGGTCAAAAC

Primers used for junction PCR of UGCG-overexpressing constitutive and inducible cell lines

Primer name	Primer seq 5'->3'
5' –Junction PCR-Fw	CTCGACATCGGCAAGGTGTG
5' –Junction PCR-Rev	ATGGAGGTCAAAACAGCGTG
3' –Junction PCR-Fw	AATGCAAAACTCTGGCTCATA
3' –Junction PCR-Rev	ATGACGTCTCCATAGAGCCCACCGCATCC
3' –Tet-On-Junction PCR-Fw	GAGAGACACCTACCACCG

Supplementary Figure S11: Percentage of mCherry-Expressing Cells (A) 72 hours after thawing and (B) Following 55 Days of stability testing.

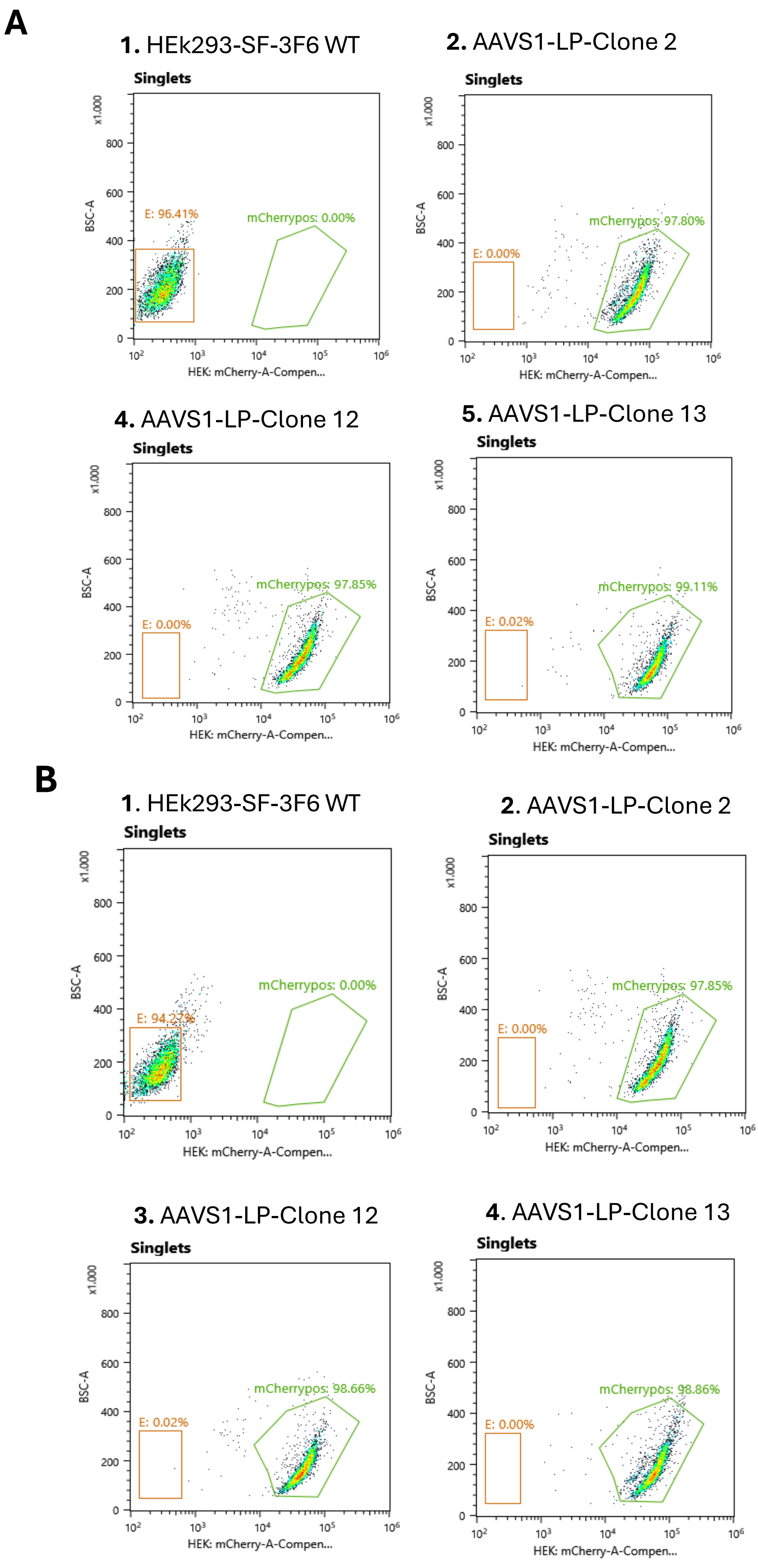

Supplement: Supplementary file 1 [file DataSheet1.pdf]
